# The Impact of Vaccination Practices in Cow–Calf Operations on Bovine Respiratory Disease Morbidity and Mortality in Beef Calves: A Rapid Scoping Review

**DOI:** 10.3390/ani16213454

**Published:** 2026-11-01

**Authors:** V. Margarita Sanguinetti, Nathan E. N. Erickson, Barbara Wilhelm, Colleen Pollock, Su Hyun Lim, Lianne McLeod, Cheryl L. Waldner

**Affiliations:** Western College of Veterinary Medicine, University of Saskatchewan, Saskatoon, SK S7N 5B4, Canadabarb.wilhelm@usask.ca (B.W.);

**Keywords:** bovine respiratory disease, beef cattle, cow–calf operations, vaccination, pre-weaning calves, maternal vaccination, scoping review, antimicrobial use

## Abstract

Background: Bovine respiratory disease (BRD) drives antimicrobial use in cow–calf herds and feedlots. This rapid scoping review evaluated vaccination of North American beef dams or pre-weaning calves against respiratory pathogens and subsequent calf health. Methods: Five databases and gray literature sources were searched for English-language North American trials and observational studies published since 2000. Data were charted in Excel and independently reviewed. Results: Twenty-eight studies reported clinical outcomes. One of seven field trials of pre-weaning calf vaccination reported pre-weaning clinical benefit; two of six reported post-weaning benefit. Four of ten calf vaccination challenge trials reported clinical differences. Two of six observational studies of dam vaccination reported beneficial pre-weaning associations. Post weaning, one dam vaccination challenge trial reduced pathogen shedding without improving clinical outcomes, while one cohort reported favorable feedlot associations. Two of the three field trials with beneficial clinical findings evaluated vaccines targeting pathogens previously associated with disease in the study herd. Observational evidence from multicomponent programs could not isolate vaccination effects. Conclusions: Evidence did not identify a consistently effective vaccination protocol. Benefits may depend on herd disease history, pathogen targets, disease pressure, and management context. Trials should evaluate clearly defined protocols in relevant settings and report absolute and relative effects with confidence intervals.

## Simple Summary

Bovine respiratory disease is an important cause of illness, death, and antimicrobial use in beef calves. This rapid scoping review examined North American research on vaccination of beef cows or pre-weaning calves against respiratory pathogens and considered calf health before and after weaning. The 28 studies reporting clinical outcomes included field trials, experimental challenge trials, and observational studies. Differences in protocols and comparators, low disease incidence, and limited sample sizes constrained interpretation. Clear health benefits were demonstrated in relatively few studies. However, two of the three field trials with positive results targeted pathogens previously associated with disease in the study herd, suggesting that herd history and diagnostic information may improve vaccine selection. Experimental challenge trials sometimes identified reduced pathogen shedding or differences in clinical scores, but these effects did not consistently correspond to less disease. Observational evaluations of multicomponent calf health programs found favorable post-weaning associations, but the contribution of vaccination could not be separated from other management practices. Evidence supporting dam vaccination was particularly limited. Laboratory outcomes were mapped separately to inform future research. Future trials should evaluate clearly defined protocols under meaningful disease pressure and report clinically relevant effect estimates with confidence intervals.

## 1. Introduction

Studies have evaluated vaccination strategies to control bovine respiratory disease (BRD) in feedlot cattle [1]. Other reviews of vaccination against BRD pathogens have included broader production classes [2,3,4,5]. A recent Canadian benchmarking study described vaccination and other calf health management practices in cow–calf herds [6], and a meta-analysis of commercially available viral vaccines pooled studies across production classes [7]. Few studies in these reviews evaluated vaccine effectiveness in North American cow–calf herds. The evidence was insufficient to determine whether vaccinating dams or pre-weaning beef calves affected pre- or post-weaning BRD morbidity or mortality. 

In Canada, treatment of BRD is a leading reason for injectable antimicrobial use (AMU) in beef cow–calf operations [8,9]. The National Animal Health Monitoring System (NAHMS) studies in the United States from 2007 to 2008 and 2017 and a 2010 western Canadian study reported mean pre-weaning BRD morbidity of 2.7–3.8% [10,11,12]. A four-year Canadian study reported pre-weaning BRD treatment of 5.3% (standard deviation (SD) = 12.6%) and mortality of 0.7% (SD = 2.8%), with substantial herd variation [13].

Because evidence supporting respiratory vaccination (i.e., vaccination against BRD pathogens, whether administered by injection or intranasally) at feedlot arrival is limited [1], vaccination in the cow–calf herd may be important for managing feedlot BRD. BRD treatment and control also drive injectable antimicrobial use in feedlots [14,15]. A 2024 feedlot surveillance program, which examined health data from 531 randomly selected closed production lots from 21 Alberta feedlots, reported that the BRD first pull morbidity rate was 15.1% [16]. The 2011 NAHMS feedlot study estimated that 16.2% (SE: 1.4%) of cattle in US feedlots with capacity of 1000 head or more were affected with respiratory disease with most treated with an injectable antimicrobial [17]. BRD therefore imposes substantial costs through production losses, treatment, and mortality [18].

Several viral agents have historically been associated with BRD and are targeted in commercial respiratory vaccines [7], including bovine respiratory syncytial virus (BRSV; *Bovine orthopneumovirus*) [19], bovine herpesvirus type 1 (BoHV-1; *Bovine alphaherpesvirus 1*, “IBR” or infectious bovine rhinotracheitis virus) [20], bovine viral diarrhea virus (BVDV-1 and BVDV-2) [21], and bovine parainfluenza virus type 3 (BPIV-3; *Bovine respirovirus-3*) [22]. Bacterial species recognized as BRD pathogens and targeted in commercially available respiratory vaccines include *Mannheimia haemolytica*, *Pasteurella multocida*, *Histophilus somni*, and *Mycoplasma bovis* [4,23]. 

Based on the results of a 2020 survey of beef producers, the use of 5-way vaccines targeting the viral BRD pathogens BRSV, BoHV-1, BVDV-1, BVDV-2 and BPIV-3 in Canadian cow–calf herds is widespread [24]. Most herds (69–92%) vaccinate dams with these multivalent vaccines, usually before breeding or during pregnancy [24,25], primarily to prevent reproductive losses associated with BoHV-1 and BVDV, such as abortion and, for BVDV, the birth of persistently infected calves [20,21]; the BRSV and BPIV-3 components of these combination products are not intended to prevent reproductive losses. This practice may also reduce calf BRD through colostral transfer of specific immunoglobulins during early immune development [26,27]. Similarly, most herds (73–92%) vaccinate pre-weaning calves with at least one dose against viral BRD pathogens [24,25]. However, there was wide variation in reported vaccination protocols against BRD pathogens in pre-weaning calves across herds [24]. Their relative effectiveness remains unknown.

Primary research has examined pre-weaning dose number [10], administration route [28,29], timing [30], dam or calf vaccination [31,32], and maternal antibody effects [33]. Researchers have also investigated the preceding questions as they pertain to priming versus subsequent booster vaccinations [28,29,34].

Synthesizing complex interventions such as vaccination requires describing their sources of complexity. The Cochrane Handbook devotes a chapter to intervention complexity [35]. The Handbook identifies three domains: intervention components, interactions among components or with context, and interactions with the wider system [35]. Vaccination protocols may involve all three domains. Cochrane therefore recommends asking not only whether an intervention works, but in which settings, circumstances, or populations it is effective. 

**Figure 1:**
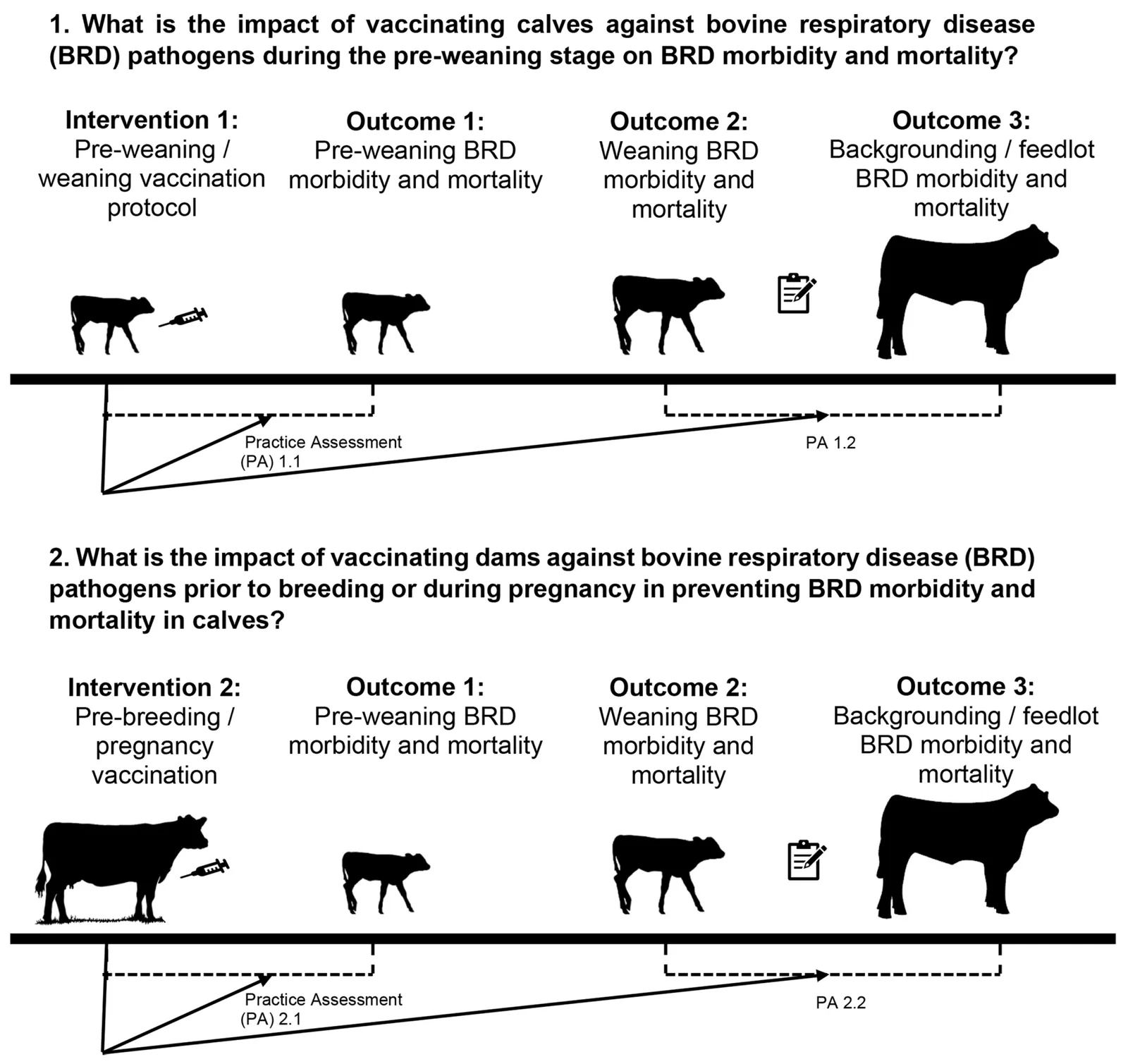
Research questions in a rapid scoping review on the impact of vaccination practices used in cow–calf operations to prevent morbidity and mortality caused by bovine respiratory disease in beef calves.

To date, which vaccination protocols in cow–calf herds are most likely to be beneficial to calf pre- and post-weaning health remains unclear. Evidence not synthesized in previous reviews [5,7,36] could narrow this gap. The research questions that guided this work were first, “What is the impact of vaccinating calves against BRD pathogens during the pre-weaning stage on BRD morbidity and mortality in calves?” and second “What is the impact of vaccinating dams against BRD pathogens prior to breeding or during pregnancy in controlling BRD morbidity and mortality in calves?” (Figure 1).

The primary objective of this rapid scoping review was to summarize the current scientific evidence on effectiveness, efficacy, and associations between dam and calf vaccination targeting BRD pathogens and their impact on calf BRD outcomes during the pre- and post-weaning stages. A secondary objective was to map the evidence and identify research gaps. 

## 2. Materials and Methods

A series of articles on evidence synthesis in veterinary medicine informed this review [37,38,39,40,41]. Cochrane rapid review and scoping review guidance was followed and reporting followed the Preferred Reporting Items for Systematic Reviews and Meta-Analysis (PRISMA) extension for scoping reviews (PRISMA-ScR) [42,43]. A completed PRISMA-ScR checklist is provided as a Supplemental File.

### 2.1. Protocol and Registration

A protocol was developed following PRISMA-ScR guidance [43]. Previous reviews of BRD vaccination under natural disease conditions informed the scope and research questions. The final protocol is available in the University of Saskatchewan Harvest repository (https://harvest.usask.ca/items/87b7930f-40ea-4f74-b7ea-bb5f72d9db2d (accessed 27 October 2026)). Screening and extraction forms are available in the protocol. Departures from the protocol were limited to the following: two reviewers (BW and CP) independently reviewed the extracted data for all included primary-outcome papers, exceeding the protocol’s planned verification procedures; post hoc comparisons of proportions were added when published group-level numerator-denominator data were available but the original report did not provide a statistical comparison; secondary-outcome mapping retained relevant conference proceedings identified through the pre-specified manual searches, whereas the primary clinical synthesis remained restricted to peer-reviewed articles and theses; secondary-outcome mapping included studies published in 2010 or later, whereas the protocol specified publication after 2010; and crude morbidity and mortality outcomes, planned for supplementary presentation, were incorporated into the main evidence tables where relevant.

### 2.2. Eligibility Criteria

Criteria specified the population, intervention, comparators, outcomes, and study designs. Eligibility criteria balanced relevance to current vaccine availability and use with rapid review resources.

#### 2.2.1. Population

Intervention populations were beef dams before breeding or during pregnancy and pre-weaning calves; outcome populations were pre- and post-weaning calves. 

#### 2.2.2. Interventions and Comparators

Eligible interventions were vaccination protocols targeting common BRD pathogens, administered by any route (e.g., parenteral or intranasal). Commercial and experimental vaccine studies required a concurrent comparator (placebo, no treatment, or an alternate vaccine). Alternative delivery technologies, such as electroporation or needle-free injection, were excluded to focus on formulation and timing. 

#### 2.2.3. Outcomes

The primary outcomes of interest were clinical measures. For natural-disease and experimental-challenge studies, outcomes included pre- and post-weaning BRD morbidity and mortality and crude morbidity and mortality. Clinical scores were summarized when reported. Post-challenge viral presence or detection was captured because pathogen shedding can affect clinical disease. Only studies reporting clinical outcomes published after 1 January 2000 were included. This review only summarizes evidence from studies reporting primary clinical outcomes. Secondary outcomes were mapped for completeness and included humoral and cellular immune biomarkers against BRD pathogens. Additional studies published in 2010 or later reporting secondary outcomes were also included. Retreatment was not a prespecified review outcome and was not included in the narrative synthesis or headline totals. Where reported, retreatment was retained only as descriptive source-level mapping. 

#### 2.2.4. Study Designs and Report Characteristics

This rapid scoping review included randomized controlled trials (RCTs) under natural or experimental BRD challenge conditions. Observational studies were included if they statistically evaluated a vaccination protocol–outcome relationship. Full texts for primary outcomes were required to be written in English and published in a peer-reviewed journal or thesis. Study sites were limited to North America.

### 2.3. Information Sources, Search Strategy, and Data Management

Five databases were searched across four platforms: CAB Abstracts via Web of Science, MEDLINE via Ovid, Web of Science, PubMed, and ProQuest (all databases, including AGRICOLA and Dissertations and Theses). An experienced librarian (SL) searched controlled vocabulary and keywords for the condition, population, and intervention. Complete database-specific search strategies, platforms, search dates, limits, and retrieval counts are provided in Supplementary Materials File S1. Searches were conducted on 10 October 2025, and updated to include *Mycoplasma bovis* on 27 October 2025. The searches were rerun on 9 September 2026; no additional studies met the inclusion criteria. Reference lists of relevant reviews were hand-searched, and additional gray literature sources were searched as described in Supplementary Materials File S1. Reports were imported into Covidence (Veritas Health Innovation, Melbourne, Australia) for deduplication and screening. Eligibility was restricted to English-language reports published after 1 January 2000.

### 2.4. Screening and Selection Process

Screening occurred in two stages. During title and abstract screening, one reviewer (VMS) screened all records, and a second reviewer (BW), blinded to the first reviewer’s decisions, independently screened a random 20% sample after a 50-record pilot exercise. Signaling questions classified records as ‘Yes,’ ‘No,’ or ‘Maybe.’ VMS and BW resolved disagreements, consulting CW and CP when required. Records classified as ‘Yes’ or ‘Maybe’ proceeded to full-text screening [44].

At the second stage, VMS and BW independently assessed a 50-report pilot to confirm consistent application of the eligibility criteria, with VMS screening the balance of the studies at this level. Signaling questions classified reports as ‘include’ or ‘exclude.’ The reviewers resolved disagreements, consulting CW and CP when required.

### 2.5. Data Collection Process

Data were extracted from studies reporting relevant clinical outcomes. A 50-record pilot reviewed the proposed extraction form. One reviewer (VMS) extracted data into Microsoft Excel (Microsoft Corporation, Redmond, WA, USA) tables, and two reviewers (BW, CP) independently reviewed the extracted data for all included papers. Investigators were not contacted to obtain or confirm data. Funding sources and funder roles for individual included studies were not synthesized because this information was considered outside the scope of the present review. Disagreements were resolved by discussion. Information was extracted at study and practice-assessment levels. A practice assessment compared a vaccination practice or protocol with an outcome within a study. Practice assessments were assigned alphanumeric codes and classified by the intervention population (dams or calves) and outcome period (pre- or post-weaning) (Figure 1). The term effectiveness described statistically significant results from RCTs under naturally occurring disease conditions, efficacy described statistically significant results from experimental challenge trials, and association described statistically significant results from observational studies. Statistical significance was defined as *p* < 0.05. Descriptive findings without statistical assessment were reported as presented.

For studies that described case counts for a specific clinical condition but did not mention BRD-associated disease, disease descriptions were assumed to be complete as reported. For example, a study reporting only lameness was coded as having no BRD-associated disease. When studies reported only crude morbidity or mortality without disease-specific information, the crude measures were retained. For studies reporting numerator-denominator values for BRD cases by vaccination group without a statistical comparison, a two-sample comparison of proportions was computed post hoc using the prtest command in StataNow 19 SE (StataCorp LLC, College Station, TX, USA). Review-author calculations were checked before inclusion and are identified in the text as post hoc.

### 2.6. Data Synthesis

Primary outcomes were summarized narratively and in descriptive tables. The narrative synthesis deliberately focused on morbidity, mortality, and treatment because these outcomes are most directly relevant to animal health and vaccination decisions in commercial herds. Studies reporting only secondary outcomes were mapped but were not included in the clinical-effectiveness synthesis. Summary tables grouped protocols by intervention population and outcome stage. Evidence was ordered by study design and disease conditions. The highest evidentiary weight was given to RCTs conducted under naturally occurring disease conditions, followed by RCTs conducted under experimental challenge conditions, and finally, observational studies, including cohort studies, case–control and cross-sectional designs [38,39,45]. 

**Figure 2:**
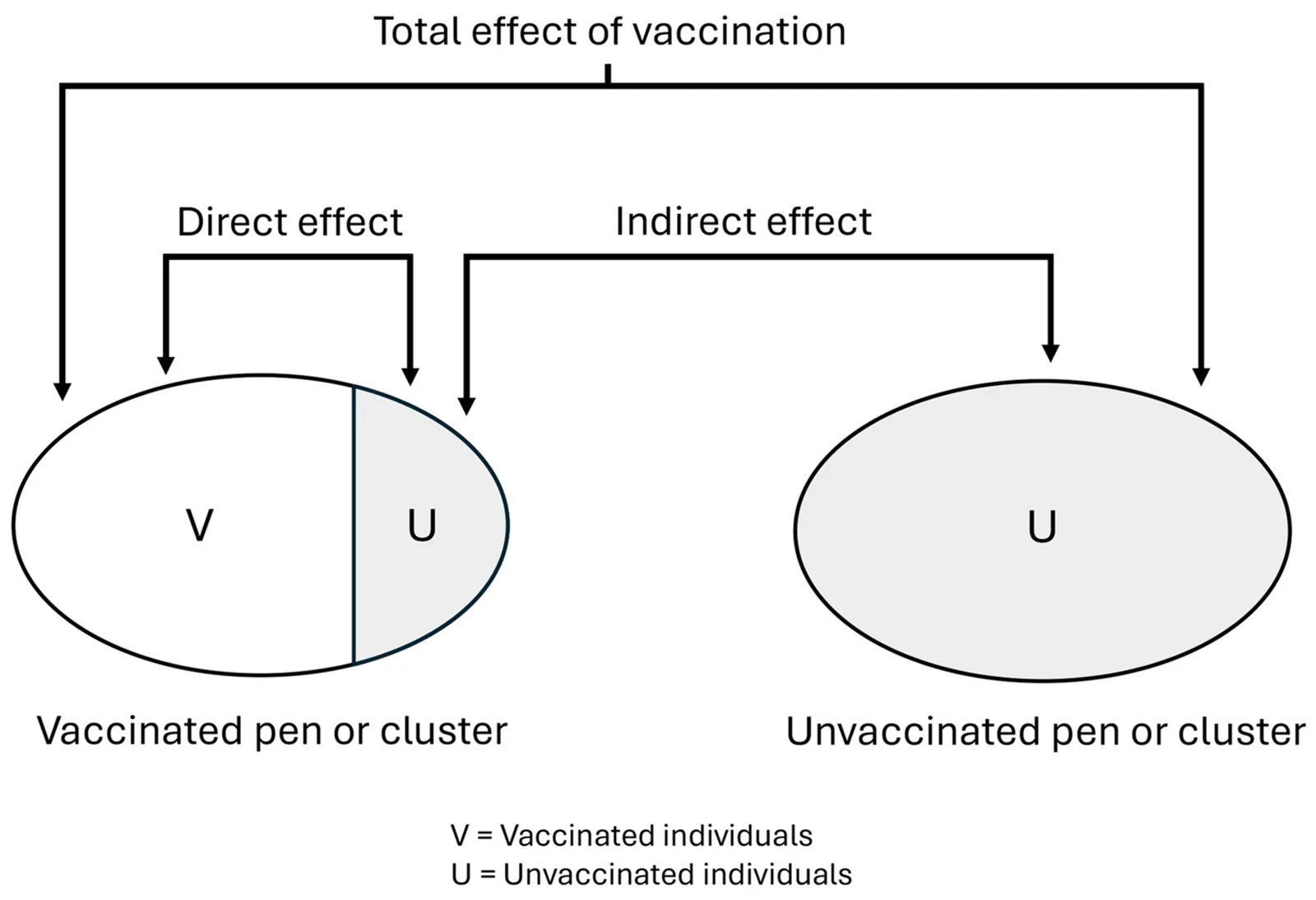
Direct and indirect effects of vaccination in pens or clusters, adapted from Knight-Jones et al. (2014) [47].

For RCTs conducted under naturally occurring disease conditions, tables showing the timing of vaccination protocols assessed and whether animal groups were commingled or not during the study were included [46]. This distinguished direct from indirect vaccine effects. Direct vaccine effects compare disease risk in similarly exposed vaccinated and unvaccinated individuals [47]. However, vaccinating some but not all members of a pen or cluster can result not only in protection of those vaccinated, but also reduced pathogen exposure and morbidity in those not vaccinated. This indirect effect occurs when vaccination reduces within-pen transmission to unvaccinated animals. Thus, partly vaccinated pens may have lower disease risk than entirely unvaccinated pens. Trials commingling vaccinated and unvaccinated animals cannot estimate indirect effects. A diagram showing the different types of vaccine effect potentially present in vaccine trials involving animals in pens or clusters is presented in Figure 2 (adapted from [47]). 

## 3. Results

**Figure 3:**
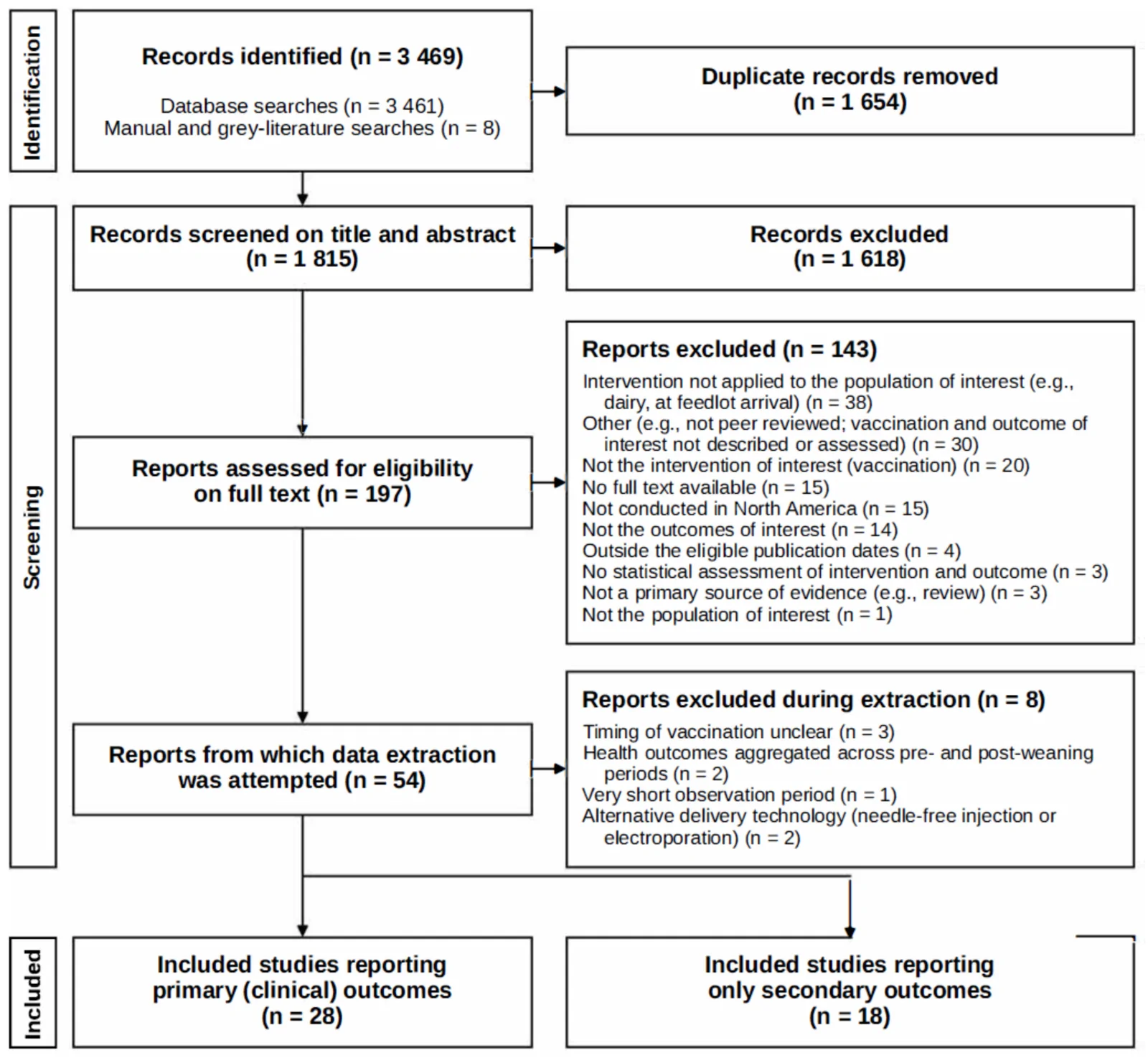
A PRISMA flowchart of a rapid scoping review on the impact of vaccination practices used in cow–calf operations to prevent morbidity and mortality caused by bovine respiratory disease in beef calves.

Database searches retrieved 3461 records, and 8 additional records were identified manually [28,48,49,50,51,52,53,54], for a total of 3469 records before deduplication. After removal of 1654 duplicate records, 1815 records underwent title and abstract screening, of which 1618 were excluded. A total of 197 reports underwent full-text screening, of which 143 were excluded. Frequent exclusion reasons were ineligible publication type, no vaccination protocol–outcome relationship, and vaccination of populations other than pre-weaning calves or dams. Data extraction was attempted for 54 reports (Figure 3).

Six studies were excluded during extraction because vaccination timing was unclear [55,56,57], outcomes combined pre- and post-weaning periods [58,59], or follow-up was very short [60]. Two studies of alternative vaccine delivery technologies, including needle-free injection and electroporation/DNA vaccination, were considered outside the review scope [61,62]. The review summarized 28 studies; Supplementary Materials File S2 maps their primary and secondary outcomes [10,24,28,29,30,31,32,33,34,48,49,50,51,54,63,64,65,66,67,68,69,70,71,72,73,74,75,76]. An additional 18 studies reported secondary outcomes only and are also listed in Supplementary Materials File S2 (Figure 3) [26,53,77,78,79,80,81,82,83,84,85,86,87,88,89,90,91,92]. Of the 28 captured studies reporting primary outcomes, 16 were conducted in Canada and 12 in the United States (Table 1).

Most studies reported multiple outcomes (Table 1). A total of 15 studies reported outcomes related to BRD morbidity or treatment, 15 related to crude or general mortality, 11 related to clinical signs or scores, 10 related to rectal temperature, 5 related to BRD mortality, and 3 related to crude or general morbidity or treatment.

### 3.1. Impact of Pre-Weaning Vaccination on Pre-Weaning Clinical Outcomes

Thirteen studies evaluated pre-weaning calf vaccination and pre-weaning health: seven natural-disease RCTs and six observational studies (two case–control; four cross-sectional) (Table 1).

#### 3.1.1. Findings from Randomized Controlled Trials Conducted Under Naturally Occurring Disease Conditions

Seven RCTs were conducted under naturally occurring disease conditions while vaccinating and assessing pre-weaning calves for health outcomes [28,29,30,33,34,50,63]. “Five-way” viral vaccines targeting BoHV-1, BVDV-1, BVDV-2, BPIV-3, and BRSV were part of the vaccine protocol administered in each of the seven RCTs [28,29,30,33,34,50,63]. Three-way vaccines targeting BoHV-1, BPIV-3, and BRSV were used in 5 studies [28,29,34,50,63]. Bovine coronavirus (BCoV) vaccines were used in 2 studies [50,63]. *M. haemolytica* vaccines were reported in 4 studies [30,33,50,63].

Studies examined prime–booster timing [28,29,30,33], administration route [28,29,34], and live versus killed vaccines [28,29].

Two recent western Canadian studies investigated the use of BCoV vaccines in two separate Alberta commercial beef herds that had previously experienced BRD associated with laboratory detection of BCoV [50,63]. The most recent study investigated the effectiveness of intranasal (IN) priming of calves 12–24 h after birth with a commercial BCoV and Rotavirus vaccine administered IN (off-label) compared to a BCoV unvaccinated control group [50]. All calves were also primed with an IN modified live viral (MLV) vaccine targeting BoHV-1, BRSV and BPIV-3 in the contralateral nostril 12–24 h after birth. Both vaccination groups were boosted with a MLV vaccine targeting BoHV-1, BRSV, BPIV-3, BVDV types 1 and 2 and a bacterin for *M. haemolytica* subcutaneously (SC) at pasture turnout or an average of 49 days of age (DOA) (SD = 7 d), and calves in the BCoV vaccine group were boosted with an intramuscular (IM) dose of the BCoV and Rotavirus vaccine at the same time [50]. The incidence of BRD reported before pasture-turnout booster vaccination was 16% and 22% in BCoV vaccinated and BCoV-unvaccinated control groups, respectively [50]. Calves in the control group that had not received IN BCoV-Rotavirus vaccine near birth were more likely than BCoV-Rotavirus IN vaccinated calves to be treated for BRD before the pasture turnout booster vaccination (odds ratio (OR) = 1.50; 95% confidence interval (CI) (1.03, 2.24), *p* = 0.048). However, within the 14 days following pasture turnout booster vaccination, the difference in BRD treatments between groups was not significant (OR = 1.72, 95% CI (0.97, 3.06), *p* = 0.06). The odds of mortality for control calves born from conceptions occurring during the dams’ second estrous cycle of the breeding season were higher (OR = 4.8; *p* = 0.001) than for vaccinated calves [50].

In an earlier study, the same research group compared the effectiveness of administering an IN priming dose of one of two commercial BCoV vaccines, or no BCoV vaccine, at ~2 months of age, boosted IM 3 weeks before weaning with the same BCoV vaccine received as the priming dose [63]. All calves, regardless of vaccination group, additionally received the farm’s usual prime and boost vaccine protocol [63]. However, no pre-weaning BRD morbidity or mortality were observed in any of the three vaccination groups before weaning [63]. In the remaining five RCTs examining pre-weaning vaccination and its impact on pre-weaning BRD morbidity or mortality, researchers reported identical rates between vaccination groups [34], nonsignificantly different rates based on a post hoc comparison [33], or no BRD-specific morbidity or mortality [28,29,30]. Among six RCTs without a significant effect, BRD-specific or crude morbidity and mortality ranged from 0% to 1.9%. Some studies did not prioritize clinical outcomes and were likely underpowered for morbidity and mortality [28,29,30]. The clinical findings for each of the seven RCTs are categorized in Table 2 as statistically significant differences, nonsignificant differences, identical rates, or no BRD events.

Pre-weaning commingling of vaccination groups was reported in each RCT except Powell et al. [30], which would limit the detectable treatment effect to direct effects only, potentially increasing the probability of reporting a nonsignificant vaccine treatment effect. Of the six RCTs that did not report a statistically significant treatment effect, three were conducted in research herds [28,29,30] and three were conducted in commercial herds [33,34,63]. Crude or general morbidity and mortality reported in some of these studies were also infrequent.

Two of seven RCTs reported desired study power or sample size calculations [50,63]. A summary of vaccination timing in these seven RCTs is presented in Supplementary Materials File S3a. An overview of primary and secondary outcomes reported across studies is presented in Supplementary Materials File S2.

No challenge studies evaluated pre-weaning calf vaccination and pre-weaning BRD.

#### 3.1.2. Findings from Observational Studies

Producer survey observational studies evaluated associations with several vaccine targets including BoHV-1, BVDV [64]; BVDV, *M. haemolytica*-*P. multocida*, or *H. somni* [24], or *M. haemolytica*-*P. multocida* [10]. Use of non-specific “respiratory vaccines” was captured in 4 studies [10,54,65,66]. Additionally, impact of the number of times calves were vaccinated with respiratory vaccines pre-weaning was captured in 1 US survey [10]. BRD morbidity was investigated in 5 studies [10,24,54,65,66]. Crude mortality was captured in 2 studies [24,64].

In a US cross-sectional survey study, results of univariable analysis indicated that in herds where respiratory vaccines were administered to pre-weaned calves, detection of BRD occurred more frequently than in herds that did not administer respiratory vaccines to their calves (*p* < 0.001) [66]. However, in the final multivariable model, pre-weaning respiratory vaccination was not clearly associated with herd-level pre-weaning BRD morbidity [66]. In a US case–control study, results of univariable analysis indicated that pre-weaning respiratory vaccination was not associated with a herd being classified as a case herd (i.e., herd-level pre-weaning BRD morbidity > 5%) [54]. In a Canadian cross-sectional survey study, respiratory vaccination was associated with a higher reported proportion of calves treated for pre-weaning BRD [65]. 

In another US cross-sectional survey, results of univariable analysis indicated higher unadjusted pre-weaning BRD incidence rates were associated with herds that administered respiratory vaccines to calves once or twice, compared to herds that did not vaccinate against BRD; however, vaccination frequency was not retained as a significant variable in the multivariable model [10]. Conversely, univariable analysis indicated lower unadjusted pre-weaning BRD incidence rates were associated with herds that administered *M. haemolytica* vaccine to pre-weaned calves compared to herds that did not vaccinate pre-weaned calves against this specific BRD pathogen; however, this variable was also not retained in the final multivariable model [10]. Another Canadian case–control study found no clear association between pre-weaning calf respiratory vaccination and crude pre-weaning mortality [64]. A recent Canadian cross-sectional study reported that suckling calves vaccinated against BVDV (OR = 2.02, 95% CI 1.17 to 3.47; *p* = 0.01) or *M. haemolytica* with or without *P. multocida* (OR = 2.31, 95% CI 1.32 to 4.07; *p* < 0.01) were associated with higher reported calf pneumonia morbidity [24]. 

These associations do not show that vaccination increased disease risk and may reflect preferential adoption by herds with previous diagnosis/treatment of BRD among pre-weaned calves [24]. Cross-sectional studies do not allow the reader to distinguish between herds in which vaccines were administered for prevention of disease and those in which vaccines were administered because of a perceived BRD problem in the herd [66]. These observational findings may reflect confounding by indication, protocol differences, or other herd factors and are not causal.

### 3.2. Impact of Pre-Weaning Vaccination on Post-Weaning Clinical Outcomes

Eighteen studies evaluated pre-weaning calf vaccination and post-weaning health: six natural-disease RCTs [28,29,30,33,34,63], ten experimental challenge trials [31,48,49,51,67,68,69,70,71,72] involving BVDV (n = 2), BoHV-1 (n = 1), BRSV (n = 2), or combined pathogens (n = 5), and two observational studies (Table 1).

#### 3.2.1. Findings from Randomized Controlled Trials Conducted Under Naturally Occurring Disease Conditions

All six RCTs included “5-way” viral targets (BoHV-1, BVDV-1, BVDV-2, BPIV-3, and BRSV) [28,29,30,33,34,63]. “Three-way” viral vaccines targeting BoHV-1, BPIV-3, and BRSV were used in 4 studies [28,29,34,63]. Bovine coronavirus (BCoV) vaccines were used in 1 study [63]. *M. haemolytica* vaccines were used in 3 studies [30,33,63]. The time period for detecting BRD morbidity in the post-weaning study period varied from as short as 2 weeks [28] to the entire period from feedlot entry to slaughter (~191 days) [33] (Table 1). Clinical findings for each RCT are summarized by outcome period in Table 2.

**Table 2:** The clinical findings of the seven randomized controlled trials (RCTs) conducted under naturally occurring disease conditions that evaluated pre-weaning calf vaccination, by outcome period and category of finding. Studies are ordered chronologically by publication year.

Study	Primary Study Aim	Vaccination Groups Compared	Pre-Weaning BRD Findings	Post-Weaning BRD Findings	Category of Clinical Finding
Kirkpatrick et al., 2008 [33] USA	Effect of age at vaccination on antibody titers and feedlot performance	Early (~67 DOA) or late (~167 DOA) priming with a 5-way MLV vaccine containing *Leptospira* and a *P. multocida*– *M. haemolytica* vaccine, with both groups boosted at weaning (~190 DOA); unvaccinated control	BRD rates did not differ significantly between groups (post hoc comparison)	Feedlot entry to slaughter (~191 d): BRD treatment 23.5% (early), 25.8% (late), and 24.6% (control), not significant; BRD mortality 0% (early), 0.8% (late), and 3.3% (control), significantly higher in control than early group	Pre-weaning: nonsignificant difference Post-weaning: significant difference (BRD mortality); nonsignificant difference (BRD treatment)
Powell et al., 2012 [30] USA	Immunologic, health, and growth responses to pentavalent MLV vaccination in the presence of maternal antibody versus a traditional regimen	Early (62 ± 17 DOA) vs. traditional (188 ± 17 DOA) priming with a 5-way MLV vaccine plus *M. haemolytica* leukotoxoid; both groups boosted at weaning (209 ± 17 DOA)	No calves treated for BRD	84 d: no calves treated for BRD	Pre-weaning: no BRD events Post-weaning: no BRD events
Erickson et al., 2020 [34] Canada	Comparison of IN and injectable routes of MLV priming for BRD control in recently weaned calves	IN vs. injectable MLV priming at ~2 months; both groups boosted with an injectable 5-way MLV vaccine at feedlot arrival	BRD morbidity 1.2% in each group	Feedlot placement to re-implantation: BRD morbidity 10% (IN) vs. 9% (injectable), *p* = 0.70; BRD mortality 1.6% vs. 0.3%, *p* = 0.10	Pre-weaning: identical rates Post-weaning: nonsignificant difference
Erickson et al., 2020 [28] Canada	BVDV-specific antibody responses to MLV vs. inactivated vaccine regimens	IN saline or IN MLV at birth, followed by injectable MLV or inactivated boosters at ~48–49 DOA and at weaning	No BRD morbidity or mortality reported ^a^	2 weeks: no BRD morbidity or mortality reported ^a^	Pre-weaning: no BRD events Post-weaning: no BRD events
Erickson et al., 2021 [29] Canada	BRSV- and BoHV-1-specific antibody responses to heterologous vs. homologous prime–boost vaccination	Injectable MLV at ~48 DOA and at weaning (homologous) vs. IN MLV at ~24 h followed by injectable MLV or inactivated boosters at ~48 DOA and at weaning (heterologous)	No BRD morbidity or mortality reported ^a^	60 d: no BRD morbidity or mortality reported ^a^	Pre-weaning: no BRD events Post-weaning: no BRD events
Erickson et al., 2024 [63] Canada	Post-weaning BRD treatment rates in calves primed and boosted with commercial BCoV vaccines vs. unvaccinated controls	IN priming (~2 months) and IM booster (3 weeks before weaning) with one of two BCoV vaccines vs. no BCoV vaccine; all calves received the farm’s usual respiratory vaccines	No BRD morbidity or mortality observed	45 d: controls had higher odds of BRD treatment than one BCoV-vaccinated group (OR = 2.2, 95% CI 1.0–4.4; *p* = 0.04); 10% of calves treated overall	Pre-weaning: no BRD events Post-weaning: significant difference
Erickson et al., 2026 [50] Canada	Pre-weaning BRD treatment rates in calves receiving commercial BCoV vaccines vs. unvaccinated controls	IN BCoV–rotavirus vaccine at 12–24 h with IM booster at pasture turnout (~49 DOA) vs. no BCoV vaccine; all calves received IN 3-way MLV at birth and SC 5-way MLV plus *M. haemolytica* at turnout	Birth to turnout: BRD 16% (vaccinated) vs. 22% (control); OR = 1.50, 95% CI 1.03–2.24; *p* = 0.048. 14 d after turnout: OR = 1.72, 95% CI 0.97–3.06; *p* = 0.06	Not assessed	Pre-weaning: significant difference (birth to turnout)

Primary study aims are paraphrased from each study’s title and stated objectives. Significant difference = statistically significant (*p* < 0.05) difference in a BRD outcome between groups; nonsignificant difference = BRD occurred but group differences were not statistically significant; identical rates = the same BRD rate was reported in each group; no BRD events = no BRD morbidity or mortality was reported in any group. ^a^ BRD case counts were not reported; consistent with the coding rule described in Section 2.5, these studies were classified as having no BRD events. BCoV = bovine coronavirus; BRD = bovine respiratory disease; CI = confidence interval; DOA = days of age; IM = intramuscular; IN = intranasal; MLV = modified-live virus; OR = odds ratio; SC = subcutaneous.

A previously described RCT where no pre-weaning BRD morbidity or mortality was observed, however, reported that unvaccinated control calves had greater odds of BRD treatment post-weaning (OR = 2.2, 95% CI (1.0, 4.4), *p* = 0.04) during the 45-day post-weaning study period, relative to calves primed IN and boosted IM with a single virus BCoV MLV vaccine in the pre-weaning period [63]. Overall, 10% of calves were treated for BRD on this Alberta farm with a history of BRD associated with BCoV, and the authors reported sample size calculations [63].

In an older US respiratory vaccine RCT, 451 calves were enrolled and assigned to either a control group or one of two vaccination groups within 48 h of birth [33]. In the first vaccination group, at the time of branding (~67 DOA) calves were primed with a bacterin containing *P. multocida* bacterial extract–*M. haemolytica* toxoid plus a “5-way” MLV vaccine which also included *Leptospira* serovars (canicola-grippotyphosa-hardjo-icterohemorrhagiae-pomona) and boosted using the same vaccines at the time of weaning (~190 DOA). In the second vaccination group, using the same vaccines, calves were primed 30 days prior to weaning (~167 DOA) and boosted at weaning (~190 DOA). The control group did not receive the respiratory vaccines at any time while at the farm of origin. A total of 361 of 451 enrolled calves were shipped to the feedlot at approximately 232 days of age, sorted by weight, and penned with calves from other sources. Initial feedlot BRD treatment rates did not differ significantly among the three vaccination groups (early vaccination, 28/119 [23.5%]; late vaccination, 31/120 [25.8%]; control, 30/122 [24.6%]). BRD mortality was higher in control calves (4/122 [3.3%]) than in calves first vaccinated at approximately 67 days of age (0/119 [0%]). BRD mortality was 1/120 among calves first vaccinated at approximately 167 days of age (0.8%) and did not differ significantly from either of the other treatment groups [33]. The study authors concluded that respiratory vaccination of pre-weaning calves had a positive effect on post-weaning clinical outcomes. They also concluded that there were inconsequential differences between the two respiratory vaccination strategies used [33].

A Canadian RCT evaluated different routes of administration (IN or injectable) for a priming dose of respiratory MLV vaccine in pre-weaned calves at approximately 2 months of age [34]. Calves in both vaccination groups were boosted with an injectable “5-way” MLV vaccine at feedlot arrival immediately after weaning. Post-weaning BRD morbidity for the IN and injectable priming vaccine groups was 10% and 9%, respectively, with no significant difference in BRD morbidity between the two vaccination groups (*p* = 0.70) from weaning to re-implantation in the feedlot [34]. The IN and injectable priming vaccine groups experienced BRD mortality rates of 1.6% and 0.3%, respectively. There was no significant difference in BRD mortality between the two vaccination groups (*p* = 0.10). The study authors noted that calves in the IN priming vaccine group had higher, but nonsignificant, odds of post-weaning BRD mortality (OR = 5.16, 95% CI (0.59, 45.2), *p* = 0.10) relative to calves in the injectable priming vaccine group [34].

Three RCTs reported no post-weaning BRD morbidity or mortality, precluding estimation of a clinical effect [28,29,30]. Two Canadian RCTs in research herds compared protocols that differed in IN MLV priming near birth and in the use of injectable MLV or inactivated vaccine doses before pasture turnout (~48–49 DOA) and at weaning [28,29]. A US RCT compared “early” (62 ± 17 DOA) with “traditional” (188 ± 17 DOA) priming with a “5-way” MLV vaccine plus *M. haemolytica* leukotoxoid, with both groups boosted at weaning (209 ± 17 DOA) [30]; no calves in either group were treated for BRD before or after weaning [30]. Vaccination groups were commingled during the 2-week [28], 60-day [29], and 84-day [30] post-weaning study periods; in the US trial, groups were kept separate before weaning [30].

Sample size calculations were reported in one RCT [63]. A summary of vaccination timing in these RCTs is presented in Supplementary Materials File S3b.

#### 3.2.2. Findings from Randomized Controlled Trials Under Experimental Disease Challenge Conditions

Across the 10 experimental challenge studies, 2 targeted BVDV, 1 BoHV-1, 2 BRSV, and 5 targeted various pathogen combinations which could include two or more of BoHV-1, BRSV, BPIV-3, or BCoV, delivered via measured nebulization (in varying doses), or close confinement with documented shedder animals [31,48,49,51,67,68,69,70,71,72]. All 10 experimental challenge studies vaccinated pre-weaned calves and assessed weaned calf health post challenge, using clinical scores which combined data on individual clinical signs such as rectal temperature, fever (yes/no), respiratory rate, or cough. Rectal temperature was reported as a separate outcome in seven studies [31,49,51,68,69,70,71], whereas BRD morbidity was reported in two studies [51,68] (Table 1). Early weaning of calves (at 2–3 months of age) was reported in 3 studies [31,69,70]. Significant differences in clinical outcomes across treatments were reported in 4 of 10 challenge trials described below, organized by the type of viral challenge.

##### Bovine Viral Diarrhea Virus (BVDV)

One [71] of two captured BVDV challenge trials [70,71] reported significant treatment group differences of a vaccination protocol on body temperature and on clinical scores. In this Canadian BVDV challenge trial, calves were IN primed with a 5-way MLV vaccine, administered “off label”, at ~24 h of age and boosted prior to pasture turnout at 54 DOA (SD = 3 d), with either an IN 3-way MLV vaccine plus an injectable MLV BVDV vaccine and *M. haemolytica* bacterin toxoid or an injectable 5-way killed-virus (KV) vaccine plus an injectable *M. haemolytica* bacterin toxoid [71]. Calves were weaned at approximately 6 months of age and were challenged with a virulent non-cytopathic BVDV strain (24515) 24 h after weaning (Day 0). 

The calves boosted with the MLV vaccines had significantly higher mean rectal temperatures on Day 7 post-challenge, than those boosted with the KV vaccine (*p* = 0.02). In comparison, calves that received KV booster vaccine had group mean rectal temperatures above the fever threshold of 39.7 °C for Days 7, 10 and 14, whereas the MLV boosted calves were above the fever threshold on Day 7 only [71]. Daily clinical scores between the two vaccination groups were not significantly different for most of the post-challenge study period, however on Day 10, calves in the IN-KV group had a significantly higher clinical score than calves in the IN-MLV group (*p* < 0.01). The study authors stated that the overall low daily clinical scores of both vaccination groups indicated that both were protected from severe clinical disease [71]. 

The other BVDV experimental challenge trial, in early weaned calves, found no significant differences in rectal temperatures or clinical scores between the unvaccinated control group and three vaccination groups [70]. Moderate-to-low maternally derived BVDV antibody levels appeared to protect all calves, including unvaccinated controls, from severe clinical disease post challenge. Two of the three evaluated vaccines significantly reduced BVDV viremia and viral shedding relative to unvaccinated controls, whereas the third vaccine did not consistently reduce viremia across the post-challenge period, although shedding among viremic calves was lower than in controls [70].

##### Bovine Herpesvirus Type 1 (BoHV-1)

One BoHV-1 experimental challenge trial was captured. This study reported significant differences in post-challenge BRD mortality and body temperature when comparing vaccinated and non-vaccinated calves [51]. Within this study, five different vaccine protocols were assessed with the first dose (V1) administered at 3–6 weeks of age and the second dose (V2) administered on the day after abrupt weaning and transport (5–6 months of age). Calves initially seronegative to BoHV-1 were either (i) not vaccinated at all (administered IN saline solution at V1 and V2), or (ii) IN primed with a 5-way MLV vaccine, administered “off label” at V1 and given IN saline at V2, or (iii) IN primed with a 5-way MLV vaccine at V1 and IN boosted using the same vaccine at V2, or (iv) administered an IN saline solution at V1 and IN primed with a MLV vaccine at V2, or (v) IN primed with a MLV vaccine at V1 and boosted SC with a MLV vaccine at V2. Bovine herpesvirus 1 challenge occurred 4 days after the second vaccination. 

Calves that received a vaccine protocol with either one or two saline solutions, had higher rectal temperatures than those receiving two MLV vaccine doses targeting viral agents, including BoHV-1, regardless of whether the route of administration of the booster vaccine was SC or IN [51]. Study investigators also reported that calves IN primed with a 5-way MLV vaccine and administered either an IN or SC booster, experienced a significant (*p* < 0.05) reduction in fever post-challenge. Also, calves in all four vaccination groups had significantly lower BRD mortality post-challenge compared to unvaccinated control calves (*p* < 0.001), and there were no significant differences in BRD mortality between the four vaccinated groups [51].

##### Bovine Respiratory Syncytial Virus (BRSV)

Two studies using BRSV challenge were identified. One study compared two different heterologous prime–boost vaccine protocols against the herd’s traditional single dose parenteral vaccine for detection of response differences [72] and the other compared vaccinated calves to nonvaccinated controls [31]. Neither of the captured BRSV challenge trials reported significant differences in clinical signs or scores across vaccination groups [31,72]. 

In a Canadian study, 18 heifer calves that had been IN primed at birth with a 3-way MLV vaccine containing BRSV, were boosted at ~2 months of age with either an injectable 5-way MLV vaccine containing BRSV or an injectable 5-way KV vaccine containing BRSV. At 6–7 months of age, all 18 calves, plus 2 controls that received only one dose of injectable 5-way MLV vaccine containing BRSV at 2 months of age, were abruptly weaned and experimentally challenged with BRSV [72].

In the other BRSV challenge study, the objective was to determine if vaccination shortly after birth, before maternal antibodies from colostrum can interfere with BRSV vaccine, results in vaccine induction of immune responses and clinical protection of young calves. US investigators compared calves primed within 6 h of birth with an IN 3-way viral vaccine or administered a saline control. All study calves were abruptly weaned at 3 months of age, transported to another facility and 5 days later experimentally challenged with BRSV [31]. 

In the US study, serology suggested that natural viral infection had occurred in both vaccination groups before experimental challenge [31].

##### Combined Viral Experimental Challenge

Overall, two out of five combined viral experimental challenge studies found differences in severe clinical signs between vaccinated and unvaccinated calves [48] or differences in clinical scores across different vaccination protocols assessed [49], while three studies did not find any differences in clinical outcomes between vaccination groups [67,68,69]. 

In a recent US trial using a combined BoHV-1 and BRSV challenge, calves were either (i) primed with a SC MLV 5-way vaccine at ~2 months of age (at branding) and boosted at ~6 months of age (one day after abrupt weaning and transport) with an IN 3-way MLV (BoHV-1, BRSV, BPIV-3) vaccine, or (ii) only received the IN 3-way MLV vaccine after weaning and transport, or (iii) received no vaccinations during the study. All calves were experimentally challenged with BRSV and BoHV-1, two days after booster vaccination [48]. The group receiving both parenteral priming and IN booster vaccinations had significantly lower odds (OR = 0.25, 95% CI (0.07, 0.96), *p* = 0.04) of developing severe clinical signs post challenge than those in the non-vaccinated control group [48].

A recent Canadian trial used a combined BRSV and BPIV-3 challenge. Calves were either (i) primed with an IN 3-way MLV vaccine at ~24 h after birth and boosted with a SC 5-way MLV plus inactivated *M. haemolytica* vaccine at 39 (±2) DOA (pasture turnout), or (ii) only primed with a SC 5-way MLV plus inactivated *M. haemolytica* vaccine at pasture turnout [49]. All calves were abruptly weaned when they were 159 ± 2 DOA, and the next day experimentally challenged with BRSV and BPIV-3, then monitored for eight days. On Day 6 post challenge, calves that were not IN-primed at birth, had significantly lower arterial oxygenation (*p* < 0.01) with all values below the cut point for hypoxemia, indicating more severe lung pathology, compared to calves that were IN primed at birth. As well, shedding of BRSV and BPIV-3 was greater on both Days 5 and 7 post-challenge (*p* < 0.01) for calves that were not IN-primed at birth compared to calves that were IN primed at birth. The calves IN primed at birth as well as parenterally boosted at pasture turnout had lower mean cumulative clinical scores (*p* = 0.002) on Day 8 post challenge than calves that were only parenterally primed at pasture turnout [49].

Three other combined viral experimental challenge trials reported nonsignificant differences in clinical outcomes across vaccination groups [67,68,69]. In the Canadian study [67], all calves were primed IN at birth with an MLV BCoV vaccine in one nostril and a MLV BRSV, BPIV-3 and BoHV-1 vaccine in the other nostril. At the time of pasture turnout (44 DOA, SD: 5 days), calves were differentially boosted by IM injection of either (i) a 5-way MLV vaccine and a MLV BCoV vaccine or (ii) a 5-way KV vaccine and a KV BCoV vaccine, abruptly weaned at 169 DOA (SD = 5 days) and the next day experimentally challenged with BCoV, BPIV-3, and BRSV coinfection. 

In one American study published in 2000 [68], all study calves were primed in the spring with an IN 2-way MLV (BoHV-1, BPIV-3) vaccine, and calves with odd-numbered ear tags were also primed with an experimental *M. haemolytica* serovar A1 bacterin toxoid. In the fall, all study calves were weaned and transported to an order buyer barn, where they were boosted with an IM 2-way MLV vaccine containing the same two viruses (BoHV-1 and BPIV-3) and calves with odd-numbered ear tags were boosted with an experimental *M. haemolytica* serovar A1 bacterin toxoid [68]. On the second day post-weaning, challenge was delivered by commingling the 101 study calves with 11 clinically sick calves from a local auction market for 3 days. Study calves were then transported 1600 km to a research feedlot and monitored for 28 days [68]. 

Another US study published in 2016 [69], evaluated the efficacy of vaccination of fall born crossbred steers at early weaning (median 95 DOA, range 60–128 DOA) with four different commercially available 5-way MLV vaccines, plus negative unvaccinated controls, 45 days prior to transportation to research facility and challenge exposure to BVDV persistently infected (PI) calves and calves acutely infected with BoHV-1. The study calves remained exposed to the PI cattle and BoHV-1 infected calves for 28 days [69]. 

Because challenge-study objectives and designs differed, results require study-specific interpretation. For example, the Erickson et al. (2024) [67] study did not compare unvaccinated and vaccinated calves; the purpose of that study was to observe variability in vaccine responses, after viral challenge, between similarly primed and differentially boosted calves. The authors interpreted the lack of a detected difference in clinical outcomes between vaccination groups as suggesting similar protection from the two prime–boost vaccine protocols [67]. In addition, the absence of a statistically significant clinical difference does not demonstrate equivalence, because an equivalence design and prespecified margin were not reported. One study reported a desired study power of 95% as well as sample size calculations as part of the description of methods [69].

#### 3.2.3. Findings from RCTs Under Disease Challenge Conditions Reporting Pathogen Isolation, Detection, or Shedding

Nine challenge trials investigating the impact of pre-weaning vaccination on post-weaning clinical outcomes also reported detection of the target pathogen in study calves after challenge [31,48,49,51,67,69,70,71,72], as did the dam vaccination challenge trial [32]. These outcomes were prespecified because pathogen shedding can influence transmission and clinical disease. The nine calf vaccination trials included two using BVDV challenge [70,71], two using BRSV challenge [31,72], one using BoHV-1 challenge [51], and four with more than one viral species in the challenge protocol [48,49,67,69]. Four used virus isolation [51,69,70,72], and five calf vaccination trials and the dam vaccination trial used real-time PCR (qPCR) or reverse-transcriptase qPCR [31,32,48,49,67,71].

Across the ten trials, target pathogens were assessed in buffy coat [71], serum [69,70], white blood cells [69,70], nasal secretions [31,32], and nasal swabs [48,49,51,67,69,70,72]. Seven trials reported the number or proportion of calves positive for the target pathogen [31,32,48,67,69,70,72], and six reported pathogen concentrations [31,49,51,67,71,72]. Outcomes were reported for single groups or group comparisons over intervals ranging from one day to the full study period, and two studies presented Kaplan–Meier plots of the cumulative probability of viral shedding [31,48]. Two studies also assayed calves for BRD pathogens other than the challenge virus and reported qPCR detection of *M. haemolytica* and *P. multocida* after challenge [31,48]. In the BoHV-1 challenge trial, 6 of 10 control calves died of fibrinous pneumonia, and *M. haemolytica* was cultured from their lungs [51].

Differences in detection or shedding between vaccinated and control calves, or between vaccination protocols, were reported in seven trials [32,49,51,67,69,70,71] but did not consistently correspond to differences in clinical outcomes. Condensed findings are presented in Table 3, and complete sampling event-level results are provided in Supplementary Materials File S4.

#### 3.2.4. Findings from Observational Cohort Studies

Both cohort studies reported beneficial associations between enrollment in defined herd-of-origin calf health programs and post-weaning BRD outcomes [73,74]. Calves sold at special auctions in Ontario that were either vaccinated twice against common BRD pathogens, castrated and dehorned, or pre-conditioned through a program that included vaccination, castration, dehorning and weaning for at least four weeks prior to sale had lower observed odds of BRD treatment during the first 28 days in the feedlot than calves sold at conventional auctions with unknown vaccination histories (OR = 0.68, 95% CI (0.50, 0.93), *p* = 0.017 and OR = 0.22, 95% CI (0.12, 0.38) *p* < 0.001, respectively) [73]. Similarly, in another cohort study, calves enrolled in two different herd-of-origin health protocols with at least a 45-day weaning period had a lower observed proportion treated for BRD during the first 28 days in the feedlot than calves with unknown herd-of-origin health and management histories (*p* = 0.009) [74]. Because these observational studies evaluated multicomponent programs, associations cannot be attributed to vaccination alone [73,74].

### 3.3. Impact of Dam Vaccination on Pre-Weaning Clinical Outcomes

Six observational studies assessed dam vaccination before breeding or during pregnancy and pre-weaning calf health [10,54,64,65,66,75]. Two were case–control [54,64] and four were cross-sectional studies [10,65,66,75] (Table 1).

#### Findings from Observational Studies

Two [65,75] of six observational studies reported beneficial associations between dam vaccination and pre-weaning calf outcomes. In a Canadian cross-sectional study, herds that did not vaccinate dams against bacterial BRD pathogens had higher observed odds of treating calves for BRD between four months of age and weaning than herds that vaccinated dams with respiratory bacterins (OR = 8.07, 95% CI 1.64, 39.7; *p* = 0.01) [65]. Another Canadian cross-sectional study estimated that herds vaccinating cows against BRD pathogens had a 1.3-percentage-point lower proportion of calf mortality than herds that did not vaccinate dams against BRD pathogens (*p* < 0.05) [75]. Residual confounding and herd-management differences may partly explain these observational associations.

An American cross-sectional study reported in their univariable analyses that herds that administered respiratory vaccines to dams, detected BRD in at least one calf in their herd more frequently, than those not giving BRD vaccines to dams (*p* < 0.001) [66]. However, in their final multivariable model, administration of respiratory vaccines to dams did not have a significant positive or negative association with the detection of BRD in pre-weaned calves [66]. The authors of this study pointed out that variables associated with detection of BRD in calves in the univariable analysis should be interpreted cautiously as they were likely confounded by differences in number of animals among herds and possibly other herd characteristics [66]. Three other observational studies reported a nonsignificant association between dam vaccination against common respiratory pathogens and occurrence of BRD in pre-weaning calves [10,54] or crude calf mortality risk [64] in the final results. A cross-sectional study published in 2013, reported that based on their univariable analyses, herds that vaccinated dams against viral respiratory pathogens were associated with higher rates of BRD in pre-weaned calves [10]. However, this variable was not included in their final multivariable model [10]. In a US case–control study published in 2018, results of univariable analysis indicated administration of respiratory vaccines to dams was not associated with a herd being classified as a case herd (i.e., herd-level pre-weaning BRD morbidity > 5%) [54]. A Canadian case–control study published in 2008, found no association between pre-breeding vaccination of dams against BVDV and BoHV-1 and calf pre-weaning mortality [64].

### 3.4. Impact of Dam Vaccination on Post-Weaning Clinical Outcomes

Two studies assessed dam vaccination and post-weaning calf health: one experimental challenge trial [32] and one retrospective cohort [76] (Table 1).

#### 3.4.1. Findings from the Randomized Controlled Trial

In an RCT, pregnant cows were either (i) primed with a multivalent KV vaccine containing BRSV at approximately 6.5–7.5 months of gestation and boosted 21 days later with the same vaccine, or (ii) received no vaccinations during the study. After calving, all study calves nursed colostrum naturally. At ~3 months of age, calves were abruptly weaned and transported to a research facility. After 5 days, they were experimentally challenged with BRSV then monitored for 28 days post challenge [32]. Under experimental BRSV challenge conditions, this RCT reported nonsignificant differences in post-challenge clinical scores, and body temperature in weaned calves born to dams vaccinated before calving with two doses of a KV vaccine containing BRSV compared to weaned calves born to unvaccinated dams [32]. Additionally, nasal swabs were collected from the study calves post-challenge and assayed for BRSV via RT-PCR. Researchers reported that the risk of shedding BRSV was significantly (*p* < 0.01) lower in calves born to vaccinated dams, and those calves also had a shorter median number of days categorized as positive during the 28-day post-challenge study period, relative to calves born to unvaccinated control dams [32]. A condensed summary of this study design and reported secondary outcomes [32] is presented in Table 3, with complete sampling event-level details provided in Supplementary Materials File S4.

#### 3.4.2. Findings from Observational Cohort Study

One retrospective cohort study reported associations between known dam vaccination history and calf post-weaning clinical outcomes in the feedlot [76]. In this study, pre-weaning vaccination status of all study calves was unknown. There were three exposure groups: (i) calves born to dams that had received a 5-way MLV vaccine before breeding (maternal viral vaccination program (MVVP) calves), (ii) calves of unknown vaccination history that were housed in the same pens as MVVP calves and (iii) calves of unknown vaccination history that were housed in pens that did not contain MVVP calves. On feedlot arrival, calves were classified as either low risk (LR), medium risk (MR) or high risk (HR) for developing BRD. Clinical outcomes included feedlot treatment rates for undifferentiated fever (UF), no fever (NF), and combined UF and NF (BRD morbidity), as well as overall and BRD-specific mortality rates. 

Among calves classified as LR for developing BRD at feedlot arrival, MVVP calves had significantly lower rates of treatment for UF, than both pen mates and non-pen mates of unknown vaccination history (*p* < 0.001). In addition, among LR calves, the BRD morbidity rate (*p* < 0.001) and BRD mortality rate (*p* = 0.001) were significantly lower in MVVP calves compared to non-pen mates of unknown vaccination history. Some favorable outcomes were also observed among pen mates of unknown history housed with calves born to vaccinated dams, suggesting possible pen-level or indirect effects [76]. Among calves classified as HR for BRD at feedlot arrival, rates of treatment for both UF and BRD morbidity (*p* < 0.001), as well as the overall (*p* = 0.009) and BRD-specific (*p* < 0.001) mortality were significantly lower in MVVP calves than in the other two comparison groups [76]. However, due to the retrospective nature of this study, it is impossible to determine if improvements in feedlot calf clinical outcomes were related directly to the maternal viral vaccination program or other unknown factors. These observational results may be affected by differences in source herd, management, risk classification, and other unmeasured factors and should not be interpreted as proof of a causal maternal vaccination effect [76].

A cross-question evidence map summarizing the available study designs, clinical signals, interpretation, and research priorities is presented in Table 4.

## 4. Discussion

The main finding of this review was that the available evidence did not identify a vaccination protocol for beef dams or pre-weaning calves that consistently reduced BRD morbidity or mortality. Two of the three natural-disease RCTs that reported clinical benefits evaluated vaccines targeting a pathogen previously diagnosed in the study herd [50,63], most other trials had low event rates and limited power (Table 2), and evidence for dam vaccination came mainly from observational studies (Table 4).

Despite increasing respiratory vaccine uptake in beef cow–calf herds [24], this rapid scoping review found relatively few studies of effects on pre- or post-weaning BRD morbidity or mortality. Across the 28 studies captured by this review, the research question underpinned by the largest number of published studies was the “impact of pre-weaning beef calf vaccination on calf post-weaning clinical outcomes”. This may reflect higher post-weaning [16,17] than pre-weaning [10,11,12,13] BRD morbidity in commercial surveys. The cost burden associated with BRD treatment and losses in older and more valuable animals and the challenges around antimicrobial treatment in animals closer to harvest, may make mitigation of post-weaning BRD a higher research priority. Post-weaning calves also tend to be located in confined feeding operations (backgrounding or feedlot pens), making it logistically easier to observe health outcomes relative to pre-weaning calves, which are often located on pasture. In addition, the numerous pens in feedlots allow for replication of vaccination groups, increasing the likelihood of detecting statistical differences between vaccination groups, if one exists.

The most studied vaccine targets in the captured RCTs using natural or experimental challenge were viral. A smaller number of studies reported vaccine protocols including *M. haemolytica* or *P. multocida*, but none reported investigation of *H. somni* vaccination. This frequent investigation of respiratory viral vaccine use in captured RCTs mirrors reported frequent use of these vaccines in beef cows and pre-weaning calves. A recent Canadian survey of 131 herds conducted in 2020 reported that in 92% of herds, beef cows and replacement heifers had received at least one viral vaccine in the past year against BoHV-1, BRSV, BPIV-3, BVDV-1, and BVDV-2; viruses commonly associated with both respiratory and reproductive diseases [24]. Also, in 92% and 78% of herds, pre-weaned calves received at least one viral vaccine against BoHV-1, BRSV, and BPIV-3 and at least one viral vaccine against BVDV-1 and BVDV-2, respectively [24]. 

In contrast, respiratory bacterin use was less frequently reported, with cows receiving one vaccine against *H. somni*, or *M. haemolytica* or *P. multocida* in 0%, 4%, and 18% of participating herds, respectively. Bacterins were reported as being used more frequently in pre-weaning calves relative to cows with pre-weaned calves receiving at least one *M. haemolytica*, *H. somni*, or *P. multocida* vaccine in 56%, 40%, and 18% of participating herds, respectively [24]. Also notable was the reported frequency of use of clostridial vaccines, with 59%, 66%, and 89% of herds reported administering at least one vaccine against Clostridial diseases to cows, replacement heifers, and pre-weaned calves, respectively [24]. Of six *H. somni* bacterin vaccines commercially available in Canada in 2020 [93], the *H. somni* antigen was more frequently combined with Clostridial bacterin/toxoids (3/6) and less frequently combined with 5-way MLV or KV respiratory viruses (2/6) [94]. Thus, the reported high use of Clostridial vaccines in beef herds in Canada, may partly explain the higher reported use of *H. somni* bacterins relative to *P. multocida* bacterins in Canadian beef herds. Given that 40% of herds reported vaccinating their pre-weaned calves against *H. somni*, we were surprised to find none of the captured RCTs investigated *H. somni* vaccination. Additional clinical trials are particularly needed for bacterial vaccine targets, including *H. somni*, which was not represented in the captured randomized evidence despite its importance as a cause of post-weaning disease [4,15,23].

The lower overall incidence of pre-weaning BRD morbidity relative to post-weaning BRD morbidity documented in Canadian and US studies of commercial beef production could also make detection of a vaccine treatment effect on pre-weaning BRD morbidity more difficult. The only RCT studying pre-weaning vaccination impacts on pre-weaning BRD to report a significant difference in BRD morbidity across vaccination groups was the recent investigation of BCoV vaccine use on an Alberta farm with a history of pre-weaning BRD associated with BCoV [50]. The incidence of BRD reported prior to pasture turnout (16% to 22% in vaccinated and control groups) was much higher than that reported by any other RCT studying this question. The remaining six RCTs reported BRD-specific or crude/general morbidity and mortality ranging from 0% to 1.9%, lower than the mean rates of pre-weaning BRD reported from US [10,11] and Canadian [12,13] surveys of commercial beef cow–calf operations. The low overall rates of these outcomes reported in those six studies could reduce their capacity to detect a treatment effect. It is possible that the studies not reporting sample size calculations while finding nonsignificant treatment differences may have lacked power to detect an effect. An alternative reporting approach for authors could be to provide an estimated treatment effect with magnitude and confidence intervals. However, in the included studies, these were rarely reported [50,63].

A recent Canadian survey of beef producers indicated that use of IN vaccines in neonatal beef calves had increased over previously reported levels [24]. Intranasal vaccines are recognized to produce a better priming immune response in calves with passively acquired maternal antibodies; however, the persistence of the response can be unpredictable and short [95]. Despite the inclusion of IN vaccines in 5 of 7 captured pre-weaning vaccination RCT protocols, there are still questions regarding optimizing their use. For example, there are gaps in our understanding of the optimal timing of IN use in beef calves and how IN vaccines might be best used in combination with other pre-weaning respiratory vaccines to reduce herd risk of pre-weaning BRD treatment. Additional research studies to evaluate vaccine protocols with sufficient study power, conducted in nursing calves across different management systems would help to fill some of these gaps. 

No observational study of pre-weaning calf vaccination and pre-weaning outcomes reported a beneficial association in its final adjusted analysis; reported findings were either null or showed higher observed disease occurrence in vaccinated herds, with substantial potential for confounding by indication. Two western Canadian studies found that herds reporting the use of pre-weaning respiratory vaccines [65], or specifically, BVDV or *M. haemolytica* vaccines [24], were more likely to report pre-weaning BRD morbidity. One explanation for this finding was that herds with a history of pre-weaning BRD had been more likely to adopt calf respiratory vaccination protocols [24]. These findings highlight the challenges of interpreting associations identified by cross-sectional studies. In contrast, other captured observational studies reported nonsignificant associations between respiratory vaccine use [54,66] or *M. haemolytica* vaccine use [10] in pre-weaning calves. These findings could reflect differences in sampling frames, heterogeneity in herd vaccination protocols and their implementation, or confounding by indication.

The impact of pre-weaning vaccination on post-weaning calf health was supported by a larger dataset of RCTs, challenge trials, and observational studies. However, only 2 of the RCTs in the group of studies investigating impact of pre-weaning vaccination on post-weaning BRD morbidity or mortality reported significant treatment differences [33,63]. The overall incidences of BRD in these two RCTs under natural disease conditions were 24.7% and 10%, respectively, with the other studies reporting incidence of BRD from 0 to 10%. Limited disease challenge reduces the ability to detect treatment differences. 

The two observational studies evaluating pre-weaning calf vaccination on post-weaning BRD both reported significantly reduced treatment in calves known to have received pre-weaning respiratory vaccines, relative to controls with unknown health histories [73,74]. BRD morbidity was also higher than in RCTs reporting nonsignificant post-weaning effects. Additionally, the Canadian study reported that calves preconditioned, that is, certified weaned for at least 28 days prior to the sale as well as vaccinated, castrated and dehorned in the pre-weaning period, had reduced post-weaning BRD treatment rates relative to those only certified vaccinated castrated and dehorned in the pre-weaning period [73]. This observed difference in BRD treatment rates may have been associated with additional weight gain or older age in the calves certified weaned prior to sale. Interestingly, the other observational study reported that heavier weight calves had significantly reduced odds of respiratory disease relative to lighter weight calves, over the entire feedlot period [74].

The research question regarding the impact of pre-weaning vaccination on post-weaning calf health was the main focus of 10 of the 11 challenge trials captured by this review. Two challenge studies described some protection from clinical disease identified in the negative control group, either due to passive immunity acquired from vaccinated dams [70] or inferred natural subclinical infection occurring prior to experimental challenge [31]. The former study aligns with the findings of observational studies [65,76] that similarly reported a significant beneficial association with dam vaccination on pre- and post-weaning BRD morbidity. These RCTs illustrate design and interpretation challenges that can reduce detection of vaccine effects in field and experimental studies. Reproducing severe clinical disease consistently by experimental inoculation is also difficult, particularly for BRSV [96]; when challenge produces only mild disease, including in unvaccinated controls, clinical differences between vaccination groups are harder to detect. Experimental challenge studies generally enrolled relatively few animals: 10 of 11 enrolled 54 or fewer calves, and several treatment groups included only 9–12 calves. Repeated measurements may support comparisons of continuous outcomes such as clinical scores, rectal temperature, or pathogen concentration, but these group sizes provide limited precision for less frequent binary outcomes such as BRD morbidity or mortality. Clinical event counts were also reported inconsistently because most studies emphasized repeated clinical scores, temperature, or pathogen detection; only two reported BRD morbidity, and one reported 6 deaths among 10 unvaccinated controls. Both significant and nonsignificant clinical findings should be interpreted in this context. Overall, the challenge literature primarily characterized clinical scores, rectal temperature, and pathogen detection rather than the frequency of clinically diagnosed BRD.

Research activity was concentrated on calf vaccination and post-weaning outcomes. No randomized trial evaluated dam vaccination and calf clinical outcomes under natural disease conditions. A smaller number of captured studies investigated the impact of dam vaccination on BRD in offspring (n = 8) [10,32,54,64,65,66,75,76] than investigated calf vaccination (n = 25) [10,24,28,29,30,31,33,34,48,49,50,51,54,63,64,65,66,67,68,69,70,71,72,73,74]. Five observational studies evaluated both calf and dam vaccination practices [10,54,64,65,66]. This likely reflects longer timelines and more complex logistics than calf vaccination studies. Of the six observational studies investigating impact of dam respiratory vaccination on pre-weaning calf BRD morbidity or mortality [10,54,64,65,66,75], two Canadian studies reported significant associations between dam vaccination and reduced odds of pre-weaning BRD morbidity [65] or crude mortality [75]. However, an earlier Canadian study and 3 US observational studies reported nonsignificant associations between dam viral vaccination and pre-weaning BRD morbidity [10,54,66] or mortality [64]. Both KV and MLV vaccines are commercially available, and producers’ choice in which type of vaccine to use may depend on season of vaccination. A recent Canadian study reported that MLV vaccines were most commonly administered to dams prior to breeding, with KV vaccines more commonly used at pregnancy testing [24]. Variation across observational studies may reflect vaccine type, timing, booster frequency, producers, and sampling frames.

RCTs conducted under natural disease conditions that investigated major research questions, including comparisons of MLV versus KV vaccines, different routes of vaccination, or different timing of priming and booster vaccination, did not report significant differences in clinical outcomes between the compared protocols [28,29,30,33,34]. Some studies may have lacked power for clinical outcomes because of logistical constraints [49] or a primary focus on nonclinical outcomes such as serology [28,29]. The importance of secondary outcomes also needs to be considered as it was common for experimental challenge studies to report differences in pathogen shedding between differentially vaccinated groups, as well as vaccine groups versus control groups. Reduced shedding could decrease pathogen exposure and contribute to herd-level protection, but the captured studies did not directly demonstrate that shedding reductions resulted in fewer clinical cases among exposed cattle. 

Several potential biases affect this rapid scoping review. Pre-weaning beef calves in North America tend to spend much of their lives on pasture, with fewer opportunities for observation and disease detection relative to post-weaned cattle in feedlots. Therefore, pre-weaning BRD morbidity may go unobserved, and BRD mortalities may suffer predation prior to detection. In addition, BRD in the captured field studies was identified clinically, and clinical diagnosis has limited sensitivity relative to lung lesions at slaughter or thoracic ultrasonography [97,98]; so, calves with mild or subclinical pneumonia may not have been detected. Similarly, three of the seven included RCTs studying pre-weaning calves were conducted in research settings, and these studies tended to have lower overall BRD rates. Thus, pre-weaning BRD frequency and vaccination effects may be difficult to estimate accurately. Additionally, most RCTs collected data on multiple outcomes, and clinical outcomes were likely not the primary focus of some of the RCTs captured. These factors may have lowered reported BRD rates and biased estimated clinical effects toward the null.

Beef respiratory vaccination meets the Cochrane definition of a complex intervention [35], with interacting components, variable outcomes, nonlinear group responses, and context-dependent tailoring. Synthesis must identify contexts in which interventions succeed, followed by evaluation of promising approaches across settings. Comparing multiple candidate protocols might better identify which works best in a specific setting than comparison with a single unvaccinated control. This is consistent with the classical epidemiological triad of agent, host, and environment: vaccination acts mainly on host immunity, whereas pathogen exposure, co-infection, and management and environmental conditions also determine whether BRD occurs, so consistent clinical effects of vaccination alone should not necessarily be expected.

In relation to the secondary objective of mapping the evidence and identifying research gaps, the evidence map (Table 4) and outcome map (Supplementary Materials File S2) highlight several gaps. No randomized trial evaluated dam vaccination under natural disease conditions, and no captured RCT evaluated *H. somni* vaccination. Field trials of pre-weaning calf vaccination were few, were often conducted in research herds with low BRD incidence, and rarely reported sample size calculations or effect estimates with confidence intervals. Challenge trials were small and emphasized clinical scores, rectal temperature, and pathogen shedding rather than BRD morbidity or mortality, and no captured RCT was designed to estimate indirect (pen- or herd-level) vaccine effects. Many studies reported antibody or other immune outcomes (Supplementary Materials File S2), but whether these responses predict clinical protection was outside the scope of this review and remains uncertain. Addressing these gaps will require adequately powered field trials under meaningful disease pressure, designs that account for clustering and indirect effects, and consistent reporting of clinically relevant outcomes.

Several limitations should be considered. The constrained timeline required rapid review methods and precluded formal quality assessment. Although title and abstract screening used blinded independent verification of a random 20% sample, full-text screening used independent dual assessment of a 50-report pilot followed by single-reviewer screening of the remaining reports, and extracted data for all included primary-outcome papers were independently reviewed by two additional reviewers. Eligibility was restricted to English-language reports published after 1 January 2000, and heterogeneity in populations, vaccine formulations, timing, comparators, outcome definitions, follow-up periods, and disease pressure limited comparisons across studies. Several observational studies evaluated multicomponent health programs, so the independent contribution of vaccination could not be isolated. A complementary synthesis of immune, virologic, and other mechanistic outcomes could help explain why some protocols altered antibody responses or pathogen shedding without detectable clinical benefits, identify candidate correlates of protection, and prioritize protocols for adequately powered clinical trials. Post hoc comparisons of proportions were conducted only when group-level numerator-denominator data were available and the original report did not provide a statistical comparison. These calculations use published aggregate data and may not account fully for clustering, randomization unit, repeated observations, covariate adjustment, or multiplicity; they are supplementary descriptive analyses, not replacements for original analyses. Low event counts also reduce the reliability and precision of large-sample proportion tests. These limitations may have influenced study classification and the apparent balance of statistically significant and nonsignificant findings. A supplementary search for studies published from October 2025 to 9 September 2026 found no additional eligible studies, underscoring the scarcity of robust clinical trials of BRD vaccination in North American cow–calf operations.

## 5. Conclusions

Few studies reported statistically significant clinical benefits of dam or pre-weaning calf vaccination for pre- or post-weaning health, and no consistently effective protocol was identified. Two of the three natural-disease RCTs with beneficial findings evaluated a vaccine targeting a pathogen previously diagnosed in the study herd, supporting protocol selection informed by herd disease history, pathogen targets, disease pressure, and management. The main shortcomings of the evidence were low event rates, small or unreported sample sizes, active-comparator designs without unvaccinated controls, limited reporting of effect estimates with confidence intervals, reliance on clinical scores and pathogen shedding in challenge trials, and the absence of randomized field trials of dam vaccination. Nonsignificant comparisons therefore do not show that protocols are equivalent. Future research should prioritize adequately powered field trials under meaningful disease pressure, including dam vaccination and bacterial vaccine targets, account for clustering and indirect effects, and report absolute and relative effects with confidence intervals.

## Figures and Tables

**Table 1:** Characteristics of the studies reporting primary clinical outcomes included in a rapid scoping review on the impact of vaccination practices used in cow–calf operations to prevent morbidity and mortality caused by bovine respiratory disease (BRD) in beef calves (n = 28). Studies are ordered chronologically by publication year and alphabetically by first author within each year.

Author, Year Published	Country, Year of Sampling	Study Design	N Recruited	Follow-Up Time	Population Characteristics	Research Question ^a^ (Infection Naturally Occurring (N), Challenge (C))	Intervention Level	Clinical Outcomes
Purdy et al., 2000 [68]	USA, N/R	RCT	101 calves	Post- weaning: 28 days	Commercial herds (4 farms) to order buyer barn; mixed-breed beef calves	1.2 (C; Sick calves introduced)	Calf	BRD morbidity/ treatment, crude/general mortality, clinical signs/scores, rectal temperature
Macartney et al., 2003 [73]	Canada, 1999–2000	Cohort	211 calf groups 12,313 calves 2-year study	Post- weaning: 28 days	Special auctions: Calves sold had received (i) primary and booster doses of KV vaccines against BoHV-1, BPIV-3, BVDV, and BRSV, and killed vaccines against *H. somni* and *M. haemolytica*. Booster given at least 2 weeks prior to auction and 2 to 4 weeks after the initial dose or (ii) the same vaccination protocol plus weaned for at least 4 weeks prior to sale. Conventional auctions: Calves sold had no specific health or management requirements	1.2 (N)	Group	BRD morbidity/ treatment, crude/general mortality
Kirkpatrick et al., 2008 [33]	USA, N/R	RCT	451 calves	Pre- weaning: 2 days of age to weaning Post- weaning: feedlot entry to slaughter (approximately 191 days)	Commercial herds (n = 2) March and April calving Study conducted in 2 years Vaccinated dams against BRD	1.1 (N), 1.2 (N)	Calf	BRD morbidity/ treatment, BRD mortality
Seeger et al., 2008 [74]	USA, 2003	Cohort	1465 calves	Post- weaning	Commercial herds; calves weighing approximately 600 lb (273 kg) were purchased at regular (n = 502) and special sales (pre-vaccinated n = 671; other pre-conditioning programs n = 292)	1.2 (N)	Calf	BRD morbidity/ treatment, crude/general mortality
Waldner and Kennedy, 2008 [64]	Canada, 2002	Case–control	30 high-, 30 low-risk herds	Pre- weaning	Commercial herds; 203 western beef herds were ranked before fall calf processing on the basis of losses reported by the herd owner. Those in the highest (>10% total loss) and lowest quartiles (<6% total loss) were eligible to be selected	1.1 (N), 2.1 (N)	Herd	Crude/ general mortality
Powell et al., 2012 [30]	USA, 2010 –2011	RCT	253 calves	Pre- weaning: 62 days of age to weaning Post- weaning: 84 days	Research herds (n = 3) in separate locations with spring and fall calving Vaccinated dams for BRD at branding Pre-weaning: separate vaccination groups Post-weaning: commingled	1.1 (N), 1.2 (N)	Calf	BRD morbidity/ treatment
Hanzlicek et al., 2013 [10]	USA, 2007 –2008	Cross-sectional	443 herds	Pre- weaning	Commercial herds selected from 24 states (which represented 28.6 million (87.8%) of the beef cows and 603,000 (79.6%) of US cow–calf operations) Median herd size = 128 (2, 847) Mean percentage of calves with BRD = 3.0 ± 7.1% and BRD rate = 1.5 ± 3.7 cases/ 10,000 calf-days at risk	1.1 (N), 2.1 (N)	Herd	BRD morbidity/ treatment
Woolums et al., 2013 [66]	USA, 2011	Cross-sectional	459 herds	Pre- weaning	Commercial herds; recruited participants from Florida, Georgia, and West Virginia, Iowa, Kansas, and Nebraska Mean herd size: 102 cows (plains) and 48 (eastern)	1.1 (N), 2.1 (N)	Herd	BRD morbidity/ treatment
Chamorro et al., 2015 [70]	USA, 2012– 2013	RCT	48 steers	Post- weaning: 28 days	Research herd Calves born in Sep-Oct 2012 with dams vaccinated against BVDV	1.2 (C; BVDV)	Calf	Clinical signs/scores, rectal temperature
Chamorro et al., 2016 [69]	USA, 2013	RCT	54 steers	Post- weaning: 28 days	Research herd Male calves born between September 1 and 1 October 2013. 200 cow herd vaccinated against BRD pathogens	1.2 (C; introduced calves with BVDV PI and calves acutely ill with BoHV-1 infection)	Calf	Clinical signs/scores, rectal temperature, crude/general mortality
Ellis et al., 2018 [72]	Canada, N/R	RCT	20 calves	Post- weaning: 14 days	Research herd Heifer calves born to multiparous Angus-cross cows	1.2 (C; BRSV)	Calf	Clinical signs/scores
Perrett et al., 2018 [76]	Canada, 2008 –2012	Cohort	MVVP group: n = 9315; PEN group: n = 463,847; NON-PEN group: n = 938,636	Post- weaning	MVVP: born to dams vaccinated pre-breeding with BVDV types 1 and 2, BoHV-1, BRSV, BPIV-3 PEN: unknown vaccination history, had MVVP pen mates NON PEN: unknown vaccination history, no MVVP pen mates	2.2 (N)	Dam	BRD morbidity/ treatment, crude/general mortality, BRD mortality, crude/general morbidity or treatment, rectal temperature
Woolums et al., 2018 [54]	USA, 2012 –2014	Case–control	30 case herds, 54 control herds	Pre- weaning	Commercial herds Herds were located in Nebraska, North Dakota, or South Dakota Mean weaning age ≥ 120 days ≥30 cows calving during spring enrollment period Case herds treated ≥5% of the calf crop for BRD prior to weaning Control herds treated <0.5% of the calf crop for BRD prior to weaning	1.1 (N), 2.1 (N)	Herd	BRD morbidity/ treatment
Hill et al., 2019 [51]	Canada, N/R	RCT	50 calves	Post- weaning: 12 days	Commercial herd Male and female Angus-cross calves 3–6 weeks of age purchased from a single herd of 750 Angus-cross multiparous cows	1.2 (C; BoHV-1)	Calf	Clinical signs/scores, rectal temperature, BRD morbidity/ treatment, BRD mortality
Erickson et al., 2020 [34]	Canada, 2017	RCT	645 calves	Pre- weaning: 2 months to weaning; Post- weaning: from feedlot placement to re-implantation	Commercial herd Calves born to multiparous cows in April–May 2017 and grazed in 7 groups Annually vaccinated dams against BRD Pre-weaning: commingled Post-weaning: commingled (also with animals outside the trial)	1.1 (N), 1.2 (N)	Calf	BRD morbidity/ treatment, BRD mortality, crude/general mortality
Erickson et al., 2020 [28]	Canada, N/R	RCT	75 calves	Pre- weaning Post- weaning: 2 weeks	Research herd Multiparous cows vaccinated against BRD Pre-weaning: commingled Post-weaning: commingled	1.1 (N), 1.2 (N)	Calf	Crude/general mortality
Erickson et al., 2021 [29]	Canada, 2017	RCT	75 calves	Pre-weaning Post- weaning: 60 days	Research herd Heifer calves born to cows that were second or greater parity Annually vaccinated dams against BRD Pre-weaning: commingled Post-weaning: commingled	1.1 (N), 1.2 (N)	Calf	Crude/general morbidity or treatment, crude/general mortality
Martínez et al., 2022 [32]	USA, 2019	RCT	40 calves	Post- weaning: 28 days	Research herd 175-head At 6.5 to 7.5 months of gestation Cows were stratified by age and assigned randomly to 2 groups Pre-weaning: commingled	2.2 (C; BRSV)	Dam	Clinical signs/scores, rectal temperature, crude/general mortality
Waldner et al., 2022 [65]	Canada, 2017– 2018	Cross-sectional	87 herds	Pre- weaning	Commercial herds Beef cow–calf herds in Alberta, Saskatchewan and Manitoba Median number of cows per herd: 185 (5th, 95th percentiles = 89, 684)	1.1 (N), 2.1 (N)	Herd	BRD morbidity/ treatment
Erickson et al., 2023 [71]	Canada, N/R	RCT	18 calves	Post- weaning: 14 days	Commercial herd April-born, male black Angus beef calves	1.2 (C; BVDV)	Calf	Clinical signs/scores, rectal temperature
Lazurko et al., 2023 [24]	Canada, 2020	Cross-sectional	131 herds	Pre- weaning	Commercial herds Cow–calf producers participating in the Canadian Cow–Calf Surveillance Network Mean number of breeding cows per herd = 193 Calving start dates varied and included January (24%), February (14%), March (25%), April (24%)	1.1 (N)	Herd	BRD morbidity/treatment, crude/general mortality
Martínez et al., 2023 [31]	USA, N/R	RCT	50 calves	Post- weaning: 28 days	Research herd Vaccinated dams against BRD before calving Pre-weaning: separate for 60 days, then commingled until weaning Post-weaning: commingled	1.2 (C; BRSV)	Calf	Clinical signs/scores, rectal temperature, crude/general mortality
Tang and Lhermie, 2023 [75]	Canada, 2017– 2018	Cross-sectional	122 herds	Pre- weaning	Commercial herds 23% had a breeding season < 63 days Spring (March-May) was the most common calving season	2.1 (N)	Herd	Crude/general mortality
Erickson et al., 2024 [63]	Canada, 2022	RCT	471 calves	Pre- weaning Post- weaning: 45 days	Commercial herd Laboratory-documented BCoV outbreaks consecutively for the previous 2 seasons Calves born April-May Pre-weaning: commingled Post-weaning: commingled	1.1 (N), 1.2 (N)	Calf	BRD morbidity/ treatment, BRD mortality, Crude/general morbidity or treatment, crude/general mortality, rectal temperature
Erickson et al., 2024 [67]	Canada, N/R	RCT	20 calves	Post- weaning: 12 days	Research herd Singleton commercial Black Angus and black Simmental heifer calves born without assistance to cows with one previous parity Vaccinated dams against BRD pathogens	1.2 (C; Combined BRSV, BPIV-3, and BCoV)	Calf	Clinical signs/scores
Martínez et al., 2024 [48]	USA, N/R	RCT	30 calves	Post- weaning: From −2 to 28 days	Research herd Crossbreed beef steers	1.2 (C; BRSV and BoHV-1)	Calf	Clinical signs/scores
Erickson et al., 2025 [49]	Canada, N/R	RCT	22 calves	Post- weaning: 8 days	Research herd April-born cross-bred heifer calves born to multiparous cows without dystocia or colostrum supplementation	1.2 (C; BRSV and BPIV-3)	Calf	Clinical signs/scores, rectal temperature
Erickson et al., 2026 [50]	Canada, 2024	RCT	887 calves	Pre- weaning: (A) Birth to pasture turnout 49 d (B) Turnout to weaning	Commercial herd Multiparous cows calved from February-May with history of BCoV-related BRD Vaccinated dams against BRD pathogens Pre-weaning: commingled	1.1 (N)	Calf	BRD morbidity/ treatment, crude/general mortality

^a^ Included studies investigated 1 or more of the following 4 research questions: 1.1. What is the impact of **pre-weaning** vaccination on **pre-weaning** BRD morbidity or mortality, or crude morbidity or mortality? 1.2. What is the impact of pre-weaning vaccination on post-weaning BRD morbidity or mortality, or crude morbidity or mortality? 2.1. What is the impact of dam vaccination on pre-weaning BRD morbidity or mortality, or crude morbidity or mortality? 2.2. What is the impact of dam vaccination on post-weaning BRD morbidity or mortality, or crude morbidity or mortality? **Abbreviations:** RCT = Randomized Controlled Trial; N/R = not reported; Crude morbidity/mortality = case definition not specific to BRD; BVDV = Bovine viral diarrhea virus; BoHV-1 = Bovine Herpesvirus-1; BPIV-3 = Bovine Parainfluenza Virus-3, BRSV = Bovine Respiratory Syncytial Virus; PI = Persistently Infected, Pre-weaning period = entire time period from birth to weaning, Post-weaning period = entire time-period from weaning to slaughter; MVVP = individual calves identified as part of Maternal viral vaccination program, i.e., their dams were known to be vaccinated, PEN = non MVVP study calf, but is pen mate of MVVP calf, NON-PEN = non-MVVP study calf with no MVVP pen mates.

**Table 3:** A descriptive summary of the key findings of 10 captured experimental challenge trials reporting viral detection or shedding. The findings are condensed to the most relevant summary or cumulative comparisons for each study; the complete sampling event-level table is provided in Supplementary Materials File S4.

Citation	Age at Vaccination (Age at Challenge)	Study Arms (Calves per Arm)	Sampling Time (Sample Type)	Estimate Type (Groups)	Calves Positive, n/N or % (CI or *p* Value)	Pathogen Load (CI or *p* Value)
**BVDV challenge studies**						
Chamorro et al., 2015 [70]	Median = 72 d (117 d)	C = Saline (n = 12 crossbred steers); T1 = BRD Shield (n = 12); T2 = Express 5 (n = 12); T3 = Bovi- Shield Gold 5 (n = 12)	Days 6–14 and Days 0–28 (WBC and serum)	Comparative (C, T1, T2, T3)	Viremia: On Day 6, C = 58.3%, T1 = 8.3%, T2 = 8.3%, and T3 = 36.4%; T1 and T2 were significantly lower than C, whereas T3 was not significantly different from C (*p* < 0.05). On Days 8, 10, and 14, T1, T2, and T3 were significantly lower than C (*p* < 0.05). Over Days 0–28, C = 11/12 (91.7%), T1 = 2/12 (16.7%), T2 = 3/12 (25.0%), and T3 = 6/11 (54.5%); C and T3 had higher proportions than T1 and T2 (*p* < 0.05).	NA
			Entire study period (Nasal Swab, viremic calves)	Comparative (C, T1, T2, T3)	Shedding among viremic calves over Days 0–28: C = 8/11 (72.7%), T1 = 0/2 (0%), T2 = 0/3 (0%), and T3 = 2/6 (33.3%); C was significantly higher than T1, T2, and T3 (*p* < 0.05).	NA
Erickson et al., 2023 [71]	24 h, mean = 54 d (SD = 3) (6 mo, after abrupt weaning)	T1 = Bovi-Shield Gold 5 IN at ~24 h, Triangle 5 + One Shot at 54 d (n = 9); T2 = Bovi-Shield Gold 5 IN at ~24 h, Inforce 3 + One Shot BVD at 54 d (n = 9)	Day 7, 10, 14 (Buffy coat)	Comparative (T1, T2)	NA	Viremia was detected in both treatment groups; T1 had significantly (*p* < 0.01) greater mean BVDV concentration on Days 7, 10, and 14 relative to T2. Magnitude not reported
			Entire 14-day study period (Buffy coat)	Descriptive	NA	Viremia in T1 increased between Days 0–3 and Days 3–7 (*p* = 0.05 and *p* = 0.01, respectively) and decreased between Days 10 and 14 (*p* = 0.03); T2 did not have an increase in viremia until Day 7 (*p* = 0.01) and had a decrease at Day 10 (*p* = 0.01). Magnitudes not reported
**BRSV challenge studies**						
Ellis et al., 2018 [72]	24 h, 2 mo (Weaned at 6–7 mo, challenged day of weaning)	C = no Inforce 3 at birth, within 24 h of birth, SC Bovi-Shield Gold FP5 at 2 mo (n = 2 Angus cross heifer calves); T1 = Inforce 3 at birth, Bovi-Shield Gold FP5 injectable at 2 mo (n = 9); T2 = Inforce 3 at birth, Triangle 5 SC at 2 mo (n = 9)	Day 0 (Deep Nasal Swab)	Descriptive	Proportion positive: C = T1 = T2 = 0	None of the calves had detectable BRSV on nasal swabs on the day of weaning/challenge (<10 PFU/mL)
			Day 5 and Day 7 (Deep Nasal Swab)	Comparative (T1, T2) and Descriptive (C)	Proportion positive: Day 5, T1 = 6/9, T2 = 5/9 (“similar”), C = 2/2; Day 7, T1 = T2 = 1/9, C = 2/2	Day 5: T1 = 30 (range 30–380) PFU/mL, T2 = <10 (range 10–250) PFU/mL, C calves = 360 and 340 PFU/mL. Day 7: T1 calf = 30 PFU/mL, T2 = 20 PFU/mL, C calves = 40 and 30 PFU/mL
			Study period (Deep Nasal swab)	Comparative (T1, T2)	Proportion positive: nonsignificant difference between T1, T2	Nonsignificant difference between T1, T2
Martínez et al., 2023 [31]	Within 6 h of birth (Abruptly weaned at approximately 3 mo of age, challenge 5 d later)	C = saline IN (n = 25 bull calves); T1 = Inforce 3 IN (n = 25 bull calves)	Days 4–10 and Days 0, 14, 21, 28 (Nasal Secretions)	Comparative (C, T1)	Proportion positive: C = T1 = 100% during Days 4–10; C = T1 = 0 on Days 0, 14, 21, 28	NA
			Study period = 28 d (Nasal Secretions)	Comparative (C, T1)	Risk of shedding: nonsignificant difference C vs. T1 (*p* = 0.60); median number of days calves positive: C = 2.17 d, T1 = 2.36 d (*p* = 0.84). Magnitude not reported	Median cycle threshold (Ct) values and interquartile ranges: nonsignificant differences C/T1
Martínez et al., 2022 [32]	Cows 6.5–7.5 months pregnant (Calves abruptly weaned at ~3 mo of age, challenge 5 d later)	C = 2 doses saline injection 21 d apart at 6.5–7.5 months of gestation (n = 20 steer calves born to unvaccinated dams); T1 = 2 doses Triangle 10 21 d apart at 6.5–7.5 months of gestation (n = 20 steer calves born to vaccinated dams)	Study period = 28 d (Nasal Secretions)	Comparative (C, T1)	Proportion positive: C = 4/20 and T1 = 1/20; median number of days calves positive: C = 2 d and T1 = 1 d. These counts are descriptive; the reported inferential comparison concerned the probability of shedding over time.	NA
			Study period = 28 d (Nasal Secretions)	Comparative (C, T1)	Risk of shedding: significantly (*p* < 0.01) lower in T1 relative to C. Magnitude not reported	NA
**BoHV-1 challenge studies**						
Hill et al., 2019 [51]	3–6 weeks; 5–6 months (abruptly weaned and transported at 5–6 months; second vaccination administered 1 day after weaning; challenged 4 days after the second vaccination)	C = IN saline 3 weeks, IN saline weaning (n = 10 Angus cross calves); T1 = IN Vista 5 SQ 3 weeks, IN saline weaning (n = 10); T2 = IN Vista 5 SQ 3 weeks, IN Vista 5 SQ weaning (n = 10); T3 = IN saline 3 weeks, IN Vista 5 SQ at weaning (n = 10); T4 = IN Vista 5 SQ 3 weeks, SC Vista 5 SQ weaning (n = 10)	Day 3 (Nasal Swabs)	Comparative (C, T1, T2, T3, T4)	NA	C had significantly increased shedding Day 3 relative to T1–T4 (*p* < 0.01). Magnitude not reported.
			Study period = 12 d (Nasal Swabs)	Comparative (C vs. T1, T2, T3, T4)	NA	Significant decreases in shedding relative to C for T2 (IN prime + IN booster) and T3 (single IN vaccination at 6 mo) (*p* < 0.01); nonsignificant decreases for T1 (single IN vaccination at 3 weeks) and T4 (IN priming with SC booster). Magnitude not reported.
			Study period = 12 d (Nasal Swabs)	Comparative (T2, T3)	NA	Reduction in shedding significantly (*p* < 0.01) greater for calves boosted IN at 6 mo (T2) relative to calves primed IN at 6 mo (T3). Magnitude not reported.
**Combined viral challenge studies**						
Chamorro et al., 2016 [69] (Challenge targets: BVDV, BoHV-1)	Median 95 d, Range 60–128 d (Challenge initiated 45 d after vaccination; median age approximately 140 d)	C = Saline injection (n = 11); T1 = BRD Shield (n = 11); T2 = Express 5 (n = 10); T3 = Bovi-Shield Gold 5 (n = 11); T4 = Viralign 6 (n = 11)	Day 0 and Days 3–28 (Serum or WBCs)	Descriptive (C, T1–T4)	BVDV viremia: Number positive= 0 in all groups at Day 0; proportion positive post challenge: C = 9/11 (Days 3–11), T1 = 1/11 (Day 6), T2 = 2/10 (Day 14), T3 = 4/11 (Days 6–10), T4 = 0 (entire study period)	NA
			Day 6, 7, 8, 9, 10, 14 (Serum or WBCs)	Comparative (C, T2, T4)	BVDV viremia: proportion positive significantly higher in C than in T2 (Days 6–10, 14) and in T4 (Days 10, 14)	NA
			Study period = 28 d (Deep Nasal Swab)	Descriptive and Comparative (C, T1–T4)	BVDV shedding: proportion positive C = 5/11 (Days 6–21), T1 = 2/11, T2 = 1/10, T3 = 3/11, T4 = 0; nonsignificant differences C vs. T1–T3 (Days 6–21); number of shedders in C significantly >T4 (*p* = 0.01). Magnitude not reported	NA
			Study period = 28 d (Serum, WBCs, Deep Nasal Swab)	Descriptive and Comparative (C, T1–T4)	BoHV-1: no positives in serum in any group; infrequent nasal swab positives (C = 1/11, T1 = 3/11, T2 = 1/10, T3 = 2/11, T4 = 1/11); proportion positive nonsignificantly different across all groups	NA
Erickson et al., 2024 [67] (Challenge targets: BCoV, BPIV-3, BRSV)	Birth and 44 d (challenged approximately 1 day after abrupt weaning at 169 d [SD = 5 d])	T1 = Calf-Guard and Inforce 3 IN at birth, ScourGuard 4KC + Triangle 5 (i.e., KV group) at 44 d (n = 9); T2 = Calf-Guard and Inforce 3 IN at birth, Calf-Guard and Bovi-Shield Gold FP5 (MLV) at 44 d (n = 11)	Day 0 (Nasal Swabs)	Descriptive (T1, T2)	BCoV: number positive nonsignificant difference T1 vs. T2; BPIV-3 and BRSV: T1 = T2 = 0	NA
			Days 5–10 (Nasal Swabs)	Comparative (T1, T2)	NA	BCoV: counts lower on Day 6 vs. Day 5 in both groups (T1: 0.3 times, *p* = 0.02; T2: 0.2 times, *p* < 0.01); on Day 10 the IN-MLV (T2) count was 0.2 times the IN-KV (T1) count (*p* = 0.05)
			Days 6–10 (Nasal Swabs)	Comparative (T1, T2)	NA	BPIV-3: IN-MLV (T2) counts higher than IN-KV (T1) on Day 6 (9.6 times, *p* = 0.02) and Day 10 (32.8 times, *p* = 0.04); T1 counts 0.2 times on Day 10 versus Day 6 (*p* = 0.01)
			Days 5–10 (Nasal Swabs)	Comparative (T1, T2)	NA	BRSV: counts fell from Day 5 to Day 6 in IN-KV (T1; *p* = 0.01) but rose in IN-MLV (T2; *p* = 0.01); on Day 6 (day of maximal shedding) the IN-MLV count was 79.0 times that in the IN-KV group (*p* < 0.01); Day 10 counts were lower than Day 6 in both groups (IN-KV *p* = 0.002; IN-MLV *p* = 0.01)
Erickson et al., 2025 [49] (Challenge targets: BRSV, BPIV-3)	Birth, 39 ± 2 d (160 d, day after abrupt weaning at 159 ± 2 d)	T1 = (prime–boost = PRB) Inforce 3 at birth, 39 ± 2 d, Bovi-Shield Gold One Shot SC (n = 9); T2 = (INJ) No vac at birth, SC (INJ) 39 ± 2 d, Bovi-Shield Gold One Shot (n = 9)	Day 5 and Day 7 (Nasal Swabs)	Comparative (T1, T2)	NA	BRSV: T2 had counts 2 times (Day 5) and 3.4 times (Day 7) greater than T1 (*p* < 0.01)
			Day 5 and Day 7 (Nasal Swabs)	Comparative (T1, T2)	NA	BPIV-3: T2 had counts 2.3 times (Day 5) and 17.1 times (Day 7) greater than T1 (*p* < 0.01)
Martínez et al., 2024 [48] (Challenge targets: BRSV, BoHV-1)	2 mo, 6 mo (3 d post weaning at ~6 mo of age)	C = saline at 2 mo, saline at 6 mo; T1 = Bovilis Vista 5 SQ at 2 mo, IN Nasalgen 3 at 6 mo; T2 = saline at 2 mo, IN Nasalgen 3 at 6 mo	Day −2 to Day 28 (Nasal Swabs)	Comparative (C, T1, T2)	BRSV: proportion positive C = T1 = T2 = 2/10 on Day −2; nonsignificant differences across sampling days; at every sampling day, a lower proportion of calves were positive to BRSV relative to BoHV-1, across all groups	NA
			Day −2 to Day 28 (Nasal Swabs)	Descriptive and Comparative (C, T1, T2)	BoHV-1: proportion positive on Day −2: C = 7/10, T1 = 8/10, T2 = 7/10; proportion positive significantly lower Day 21, 28 relative to Day −2, 5, 7, 10	NA
			Study period = 30 d, i.e., Day −2 to Day 28 (Nasal Swabs)	Comparative (C, T1, T2)	Day −2 to 28, the risk of shedding BRSV and BoHV-1 was not significantly different across time	NA

Where original publications reported significance testing but did not provide effect-size estimates, magnitude was recorded as not reported. Note: For Erickson et al., 2025 [49], 22 calves were initially recruited, but 4 were excluded/lost to follow-up prior to the viral challenge phase, resulting in 18 calves (9 per arm) analyzed for secondary outcomes. For Chamorro et al., 2015 [70], percentages were recalculated from the reported counts, and T3 included 11 calves at challenge because one calf was euthanized before challenge. BCoV = Bovine coronavirus; BoHV-1 = Bovine herpesvirus-1; BPIV-3 = Bovine parainfluenzavirus-3; BRSV = Bovine Respiratory Syncytial Virus; BVDV = Bovine viral diarrhea virus. SD = Standard deviation; d = Calendar day; hr = hour; mo = month; C = Control group; T1, T2, … = Treatment groups; CI = confidence interval; IN = intranasal; SC = subcutaneous; MLV = modified-live virus; KV = killed (inactivated) virus; WBC = White Blood Cells; NA = Not applicable. Commercial vaccines named in the table (antigens; vaccine type): BRD Shield, Express 5, Bovi-Shield Gold 5, and Bovi-Shield Gold FP5 (BoHV-1, BVDV-1, BVDV-2, BPIV-3, and BRSV; MLV); Bovi-Shield Gold One Shot (the same five viruses, MLV, plus *M. haemolytica* toxoid); Bovilis Vista 5 SQ (BoHV-1, BVDV-1, BVDV-2, BPIV-3, and BRSV; MLV; given IN and SC by Hill et al., 2019 [51]); Viralign 6 (BoHV-1, BVDV-1a, BVDV-1b, BVDV-2, BPIV-3, and BRSV; MLV); Inforce 3 and Nasalgen 3 (BoHV-1, BPIV-3, and BRSV; MLV for intranasal use); Triangle 5 (BoHV-1, BVDV-1, BVDV-2, BPIV-3, and BRSV; KV); Triangle 10 (the same five viruses, KV, plus five *Leptospira* serovars); One Shot (*M. haemolytica* toxoid); One Shot BVD (BVDV-1 and BVDV-2, MLV, plus *M. haemolytica* toxoid); Calf-Guard (bovine rotavirus and BCoV; MLV); and ScourGuard 4KC (bovine rotavirus and BCoV, KV, plus *Escherichia coli* K99 bacterin and *Clostridium perfringens* type C toxoid).

**Table 4:** Evidence map of study designs, clinical signals, interpretation, and research priorities for four review questions.

Research Question	Evidence Available	Clinical Signal	Interpretation and Research Priority
Calf vaccination → pre-weaning outcomes	Seven natural-disease RCTs; six observational studies; no challenge trials [10,24,28,29,30,33,34,50,54,63,64,65,66].	One of seven RCTs reported benefit [50]. No beneficial association was retained in final adjusted observational analyses [10,24,54,64,65,66].	Low event rates and limited power constrained interpretation. Priorities are adequately powered field trials under meaningful disease pressure and evaluation of context-specific protocols.
Calf vaccination → post-weaning outcomes	Six natural-disease RCTs; ten challenge trials; two observational cohorts [28,29,30,31,33,34,48,49,51,63,67,68,69,70,71,72,73,74].	Two of six field RCTs [33,63] and four of ten challenge trials [48,49,51,71] reported clinical differences. Both cohorts reported favorable associations [73,74].	Benefits were context-dependent. Challenge trials emphasized scores and pathogen detection; cohort findings could not isolate vaccination from other program components. Priorities are adequately powered field trials, appropriate active or unvaccinated comparators, and effect estimates with confidence intervals.
Dam vaccination → pre-weaning outcomes	Six observational studies; no RCTs [10,54,64,65,66,75].	Two of six observational studies reported beneficial associations [65,75].	No causal evidence was available under natural disease conditions. Priorities are randomized field studies evaluating vaccine type, timing, and clinically important calf outcomes.
Dam vaccination → post-weaning outcomes	One challenge RCT and one retrospective cohort; no natural-disease RCTs [32,76].	The challenge trial reduced shedding without clinical benefit [32]; the cohort reported favorable associations [76].	Evidence is sparse and does not establish clinical effectiveness. Priorities are evaluation of clinical and transmission outcomes under natural disease conditions and clearer separation of maternal vaccination effects from herd and calf management.

Abbreviations: BRD, bovine respiratory disease; RCT, randomized controlled trial.

## Data Availability

All data generated during this review are contained within the article and its Supplementary Materials. The review protocol is available in the University of Saskatchewan HARVEST repository at https://harvest.usask.ca/items/87b7930f-40ea-4f74-b7ea-bb5f72d9db2d accessed on 27 October 2026).

## Supplementary Materials

The following supporting information can be downloaded at: https://www.mdpi.com/article/10.3390/ani16213454/s1, Supplementary Materials File S1: Supplementary_Material_1.docx, Complete Search Strategies; Supplementary Materials File S2: Supplementary_Material_2.xlsx, Outcomes reported in studies contributing primary or secondary evidence to the rapid scoping review; Supplementary Materials File S3: Supplementary_Material_3.docx, Timing of vaccine administration in randomized controlled trials assessing the effectiveness of calf pre-weaning vaccination on pre- and post-weaning bovine respiratory disease and crude health outcomes (Supplementary Materials File S3a,b); Supplementary Materials File S4: Supplementary_Material_4.docx, Descriptive summary of findings of 10 captured experimental challenge trials reporting viral detection or shedding (complete version of manuscript Table 3); and Supplementary Materials File S5: PRISMA-ScR_Checklist.docx, completed PRISMA-ScR checklist.
