# Supplementary material for: The Impact of Vaccination Practices in Cow–Calf Operations on Bovine Respiratory Disease Morbidity and Mortality in Beef Calves: A Rapid Scoping Review
Source: Animals (Basel). 2026 Nov 1;16(21):3454. doi: 10.3390/ani16213454 (PMC13761806; doi:10.3390/ani16213454)
Supplement: Supplementary file 1 [file animals-16-03454-s001.zip › SupplementaryMaterialS1.pdf]

Impact of vaccination practices in cow-calf operations on bovine respiratory disease morbidity and mortality in beef calves: a rapid scoping review

V. Margarita Sanguinetti, Nathan E.N. Erickson, Barbara Wilhelm, Colleen Pollock, Su Hyun Lim, Lianne McLeod, Cheryl L. Waldner

Supplementary Material S1. Complete Search Strategies

The primary database searches were conducted on October 10, 2025. An additional search for *Mycoplasma bovis* was conducted on October 27, 2025. Complete database-specific strategies, date limits, and retrieval counts are reported below.
The searches were rerun on September 9, 2026; no additional studies met the inclusion criteria.

CAB Abstracts records were retrieved through the Web of Science All Databases searches, which include CAB Abstracts; the CAB Abstracts-specific search string is shown for completeness, and its records are counted within the Web of Science totals. The per-database counts below total 3,461 records (3,067 from the main search and 394 from the Mycoplasma bovis search).

Search concepts

1. Condition/Disease: pneumonia OR bovine respiratory disease OR respiratory OR shipping fever OR BRD OR bovine coronavirus OR bovine rhinotracheitis OR bovine herpes* OR bovine respiratory syncytial virus OR BRSV OR Mannheimia haemolytica OR parainfluenza 3 OR parainfluenza virus 3 OR torovirus OR Pasteurella multocida OR BVDV OR BVD OR "Bovine Viral Diarrhea"
2. Population
    a. Beef OR suckler OR feedlot* AND
    b. cow OR cows OR calf OR calves OR cattle OR dam OR dams OR heifer* OR cow-calf OR pairs OR steers OR feeder
3. Intervention: vaccin* OR immuniz* OR survey

CAB Abstracts (via Web of Science)

(de:("Bovine viral diarrhea virus 2" OR "Bovine viral diarrhea virus 1" OR "Bovine respiratory syncytial virus" OR "Bovine coronavirus" OR "Bovine herpesvirus 1" OR "Bovine herpesvirus 5" OR "Bovine torovirus" OR "Mannheimia haemolytica" OR "Pasteurella multocida" OR "Pasteurella multocida subsp. gallicida" OR "Pasteurella multocida subsp. multocida" OR "Pasteurella multocida subsp. septica" OR "Bovine parainfluenza virus 3") OR Title:(BRD OR BVD OR "bovine coronavirus" OR "bovine rhinotracheitis" OR "bovine respiratory disease" OR "bovine herpes*" OR "bovine respiratory syncytial virus" OR BRSV OR "mannheimia haemolytica" OR "parainfluenza 3" OR "parainfluenza virus 3" OR torovirus OR "Pasteurella multocida" OR BVDV OR "bovine viral diarrhea" OR pneumonia OR "shipping fever" OR respiratory) OR ab:(BRD OR BVD OR "bovine coronavirus" OR "bovine rhinotracheitis" OR "bovine respiratory disease" OR "bovine herpes*" OR "bovine respiratory syncytial virus" OR BRSV OR "mannheimia haemolytica" OR "parainfluenza 3" OR "parainfluenza virus 3" OR torovirus OR "Pasteurella multocida" OR BVDV OR "bovine viral diarrhea" OR "shipping fever" OR respiratory OR pneumonia)) AND (de:("beef cattle" OR "suckler herds" OR "feedlot*") OR Title:(beef OR suckler* OR feedlot*) OR ab:(beef OR suckler* OR feedlot*)) AND (de:(calves OR "calf disease") OR Title:(cow OR cows OR calf OR calves OR cattle OR dam OR dams OR heifer* OR cow-calf OR pairs OR steers OR feeder) OR ab:(cow OR cows OR calf OR calves OR cattle OR dam OR dams OR heifer* OR cow-calf OR pairs OR steers OR feeder)) AND (de:(vaccine OR vaccination OR immunization) OR Title:(vaccin* OR immuniz* OR survey) OR ab:(vaccin* OR immuniz* OR survey))

MEDLINE via Ovid

Timespan: Publication Date 2000 – 2027

Total records retrieved: 398 (October 10, 2025)

1	exp Bovine Respiratory Disease Complex/ OR exp Diarrhea Viruses, Bovine Viral/	3869
2	exp Coronavirus, Bovine/	391
3	exp Herpesvirus 1, Bovine/	2376
4	exp Torovirus Infections/ OR exp Torovirus/	115
5	exp Mannheimia haemolytica/	1115
6	exp Pasteurella multocida/	2317
7	exp Parainfluenza Virus 3, Bovine/	142
8	(BRD OR BVD OR bovine coronavirus OR bovine rhinotracheitis OR bovine respiratory disease OR bovine herpes* OR bovine respiratory syncytial virus OR BRSV OR mannheimia haemolytica OR parainfluenza 3 OR parainfluenza virus 3 OR torovirus OR Pasteurella multocida OR BVDV OR "bovine viral diarrhea" OR pneumonia OR "shipping fever" OR respiratory).kf,tw,sh.	805606
9	1 OR 2 OR 3 OR 4 OR 5 OR 6 OR 7 OR 8	806243
10	(beef OR suckler* OR feedlot*).kf,tw,sh.	37499
11	(cow OR cows OR calf OR calves OR cattle OR dam OR dams OR heifer* OR cow-calf OR pairs OR steers OR feeder).kf,tw,sh.	727156
12	exp Vaccines/	302527
13	exp Vaccination/	121876
14	exp Immunization/	227312
15	(vaccin* OR immuniz* OR survey).kf,tw,sh.	1402525
16	12 OR 13 OR 14 OR 15	1462680
17	9 and 10 and 11 and 16	515
18	limit 17 to yr="2000 - 2027"	398

Web of Science

Web of Science All Databases, including CAB Abstracts

Timespan: Publication Date 2000-01-01 – 2027-12-31

Total records retrieved: 1,439 (October 10, 2025)

TS=(BRD OR BVD OR "bovine coronavirus" OR "bovine rhinotracheitis" OR "bovine respiratory disease" OR "bovine herpes*" OR "bovine respiratory syncytial virus" OR BRSV OR "mannheimia haemolytica" OR "parainfluenza 3" OR "parainfluenza virus 3" OR torovirus OR "Pasteurella multocida" OR BVDV OR "bovine viral diarrhea" OR pneumonia OR "shipping fever" OR respiratory) AND TS=(beef OR suckler* OR feedlot*) AND TS=(cow OR cows OR calf OR calves OR cattle OR dam OR dams OR heifer* OR cow-calf OR pairs OR steers OR feeder) AND TS=(vaccin* OR immuniz* OR survey)

ProQuest

All Databases (including AGRICOLA; excluding EconLit)

Timespan: Publication Date 2000-01-01 – 2027-12-31

Total records retrieved: 815 (October 10, 2025)

noft(BRD OR BVD OR "bovine coronavirus" OR "bovine rhinotracheitis" OR "bovine respiratory disease" OR "bovine herpes*" OR "bovine respiratory syncytial virus" OR BRSV OR "mannheimia haemolytica" OR "parainfluenza 3" OR "parainfluenza virus 3" OR torovirus OR "Pasteurella multocida" OR BVDV OR "bovine viral diarrhea" OR pneumonia OR "shipping fever" OR respiratory) AND noft(beef OR suckler* OR feedlot*) AND noft(calf OR calves OR cow OR cows OR cattle OR dam OR dams OR heifer* OR cow-calf OR pairs OR steers OR feeder) AND noft(vaccin* OR immuniz* OR survey)

PubMed

Timespan: Publication Date 2000-01-01 – 2027-12-31

Total records retrieved: 415 (October 10, 2025)

("BRD"[All Fields] OR "BVD"[All Fields] OR "bovine coronavirus"[All Fields] OR "bovine rhinotracheitis"[All Fields] OR "bovine respiratory disease"[All Fields] OR "bovine herpes"[All Fields] OR "bovine respiratory syncytial virus"[All Fields] OR "BRSV"[All Fields] OR "mannheimia haemolytica"[All Fields] OR "parainfluenza 3"[All Fields] OR "parainfluenza virus 3"[All Fields] OR ("torovirus"[MeSH Terms] OR "torovirus"[All Fields] OR "toroviruses"[All Fields]) OR "Pasteurella multocida"[All Fields] OR "BVDV"[All Fields] OR "bovine viral diarrhea"[All Fields] OR "pneumonia"[All Fields] OR "shipping fever"[All Fields] OR "respiratory"[All Fields]) AND ("beef"[All Fields] OR "suckler*"[All Fields] OR "feedlot*"[All Fields]) AND ("calf"[All Fields] OR ("calve"[All Fields] OR "calved"[All Fields] OR "calves"[All Fields] OR "calving"[All Fields] OR "calvings"[All Fields]) OR ("cattle"[MeSH Terms] OR "cattle"[All Fields] OR "cow"[All Fields]) OR ("cattle"[MeSH Terms] OR "cattle"[All Fields] OR "cows"[All Fields]) OR ("cattle"[MeSH Terms] OR "cattle"[All Fields] OR "cattle s"[All Fields] OR "cattles"[All Fields]) OR "dam"[All Fields] OR "dams"[All Fields] OR "heifer*"[All Fields] OR "cow-calf"[All Fields] OR "pairs"[All Fields] OR "steers"[All Fields] OR "feeder"[All Fields]) AND ("vaccin*"[All Fields] OR "immuniz*"[All Fields] OR "survey"[All Fields])

Additional search: *Mycoplasma bovis* (October 27, 2025)

Includes:

1. Condition/disease: "Mycoplasma bovis," "M bovis," or "M. bovis"
2. Population
   a. Beef OR suckler OR feedlot* AND
   b. cow OR cows OR calf OR calves OR cattle OR dam OR dams OR heifer* OR cow-calf OR pairs OR steers OR feeder
3. Intervention: vaccin* OR immuniz* OR survey

MEDLINE via Ovid

https://cyber.usask.ca/login?url=http://ovidsp.ovid.com/ovidweb.cgi?T=JS&NEWS=N&PAGE=main&SHAREDSEARCHID=5K0EJG8u13KccNPBCakkLuCpMY147z6mISXK3fwx9BJShe290IiceoMk2hdHVVzYa

Timespan: Publication Date 2000 – 2027

Total records retrieved: 42 (October 27, 2025)

1	exp Mycoplasma bovis/	488
2	("Mycoplasma bovis" OR "M Bovis" OR "M. bovis").kf,tw,sh.	5913
3	1 OR 2	5913
4	(beef OR suckler* OR feedlot*).kf,tw,sh.	37572
5	(cow OR cows OR calf OR calves OR cattle OR dam OR dams OR heifer* OR cow-calf OR pairs OR steers OR feeder).kf,tw,sh.	728161
6	exp Vaccines/	302888
7	exp Vaccination/	122081
8	exp Immunization/	227661
9	(vaccin* OR immuniz* OR survey).kf,tw,sh.	1405880
10	6 OR 7 OR 8 OR 9	1466133
11	3 and 4 and 5 and 10	49
12	limit 11 to yr="2000 - 2027"	42

Web of Science

https://www.webofscience.com/wos/alldb/summary/927b367d-b4e3-46d6-9c7a-d6ee0f3dc019-0184437836/relevance/1

Web of Science All Databases, including CAB Abstracts

Timespan: Publication Date 2000-01-01 – 2027-12-31

Total records retrieved: 177 (October 27, 2025)

TS=("mycoplasma bovis" OR "M bovis" OR "M. bovis") AND TS=(beef OR suckler* OR feedlot*) AND TS=(cow OR cows OR calf OR calves OR cattle OR dam OR dams OR heifer* OR cow-calf OR pairs OR steers OR feeder) AND TS=(vaccin* OR immuniz* OR survey)

ProQuest

All Databases (including AGRICOLA; excluding EconLit)

Timespan: Publication Date 2000-01-01 – 2027-12-31

Total records retrieved: 126 (October 27, 2025)

noft("mycoplasma bovis" OR "M bovis" OR "M. bovis") AND noft(beef OR suckler* OR feedlot*) AND noft(calf OR calves OR cow OR cows OR cattle OR dam OR dams OR heifer* OR cow-calf OR pairs OR steers OR feeder) AND noft(vaccin* OR immuniz* OR survey)

PubMed

Timespan: Publication Date 2000-01-01 – 2027-12-31

Total records retrieved: 49 (October 27, 2025)

("mycoplasma bovis"[All Fields] OR "M bovis"[All Fields] OR "M. bovis"[All Fields]) AND ("beef"[All Fields] OR "suckler*"[All Fields] OR "feedlot*"[All Fields]) AND ("calf"[All Fields] OR ("calve"[All Fields] OR "calved"[All Fields] OR "calves"[All Fields] OR "calving"[All Fields] OR "calvings"[All Fields]) OR ("cattle"[MeSH Terms] OR "cattle"[All Fields] OR "cow"[All Fields]) OR ("cattle"[MeSH Terms] OR "cattle"[All Fields] OR "cows"[All Fields]) OR ("cattle"[MeSH Terms] OR "cattle"[All Fields] OR "cattle s"[All Fields] OR "cattles"[All Fields]) OR "dam"[All Fields] OR "dams"[All Fields] OR "heifer*"[All Fields] OR "cow-calf"[All Fields] OR "pairs"[All Fields] OR "steers"[All Fields] OR "feeder"[All Fields]) AND ("vaccin*"[All Fields] OR "immuniz*"[All Fields] OR "survey"[All Fields])

Additional manual searches

The Bovine Practitioner, 2000 to the search date
American Association of Bovine Practitioners annual conference proceedings, 2000 to the search date
Academy of Veterinary Consultants meeting publications, 2000 to the search date

Bovine Respiratory Disease Symposium publications, 2000 to the search date
Google Scholar searches using the electronic search terms
Reference lists of the three identified systematic and scoping reviews (references 5, 7, and 36)

Abbreviations

BRD, bovine respiratory disease; BRSV, bovine respiratory syncytial virus; BVD, bovine viral diarrhea; BVDV, bovine viral diarrhea virus; MEDLINE via Ovid, Medical Literature Analysis and Retrieval System Online.
