# Supplementary material for: The Impact of Vaccination Practices in Cow–Calf Operations on Bovine Respiratory Disease Morbidity and Mortality in Beef Calves: A Rapid Scoping Review
Source: Animals (Basel). 2026 Nov 1;16(21):3454. doi: 10.3390/ani16213454 (PMC13761806; doi:10.3390/ani16213454)
Supplement: Supplementary file 1 [file animals-16-03454-s001.zip › SupplementaryMaterialS3.pdf]

Impact of vaccination practices in cow-calf operations on bovine respiratory disease morbidity and mortality in beef calves: a rapid scoping review

V. Margarita Sanguinetti, Nathan E.N. Erickson, Barbara Wilhelm, Colleen Pollock, Su Hyun Lim, Lianne McLeod, Cheryl L. Waldner

Supplementary Material S3a. Timing of vaccine administration in seven randomized controlled trials assessing the effectiveness of calf pre-weaning vaccination on pre-weaning bovine respiratory disease and crude health outcomes.

	**Weaning age**	**Priming**		**Booster 1**		**Booster 2**
**Study**		**<5 months**	**>5 months**	**<5 months**	**>5 months**	**>5 months**
Erickson et al., 2020a [34]	5–6 months	2 months			Upon arrival at the feedlot immediately after weaning, approximately 5–6 months of age	
Erickson et al., 2020b [28]	185 d	24 h		48 d		185 d, at weaning
Erickson et al., 2021 [29]	6 months	24 h or 48 d, depending on treatment group		48 d, depending on treatment group		At weaning, 6 months
Erickson et al., 2024a [63]	141–171 d	1–2 months		4–5 months		
Erickson et al., 2026 [50]	258 d	12–24 h		49 d		
Kirkpatrick et al., 2008 [33]	190 d	67 d in the early-vaccination group	167 d in the late-vaccination group		190 d, at weaning, in both groups	
Powell et al., 2012 [30]	Mean age = 209 ± 17 d	62 ± 17 d in the early-vaccination group	188 ± 17 d in the traditional-vaccination group		Mean 209 ± 17 d, at weaning, in both groups	

Note: Ages appearing in separate priming columns can represent alternative study groups rather than sequential doses administered to the same calves. For Erickson et al. (2021), calves were primed at either 24 h or 48 d, depending on treatment group; only groups primed at 24 h received a booster at 48 d. A blank cell indicates that no dose was administered at that stage.

Supplementary Material S3b. Timing of vaccine administration in six randomized controlled trials assessing the effectiveness of calf pre-weaning vaccination on post-weaning bovine respiratory disease and crude health outcomes.

	**Weaning age**	**Priming**		**Booster 1**		**Booster 2**
**Study**		**<5 months**	**>5 months**	**<5 months**	**>5 months**	**>5 months**
Erickson et al., 2020a [34]	5–6 months	2 months			Upon arrival at the feedlot immediately after weaning, approximately 5–6 months	
Erickson et al., 2020b [28]	185 d	24 h		48 d		185 d, at weaning
Erickson et al., 2021 [29]	6 months	24 h or 48 d, depending on treatment group		48 d, depending on treatment group		At weaning, 6 months
Erickson et al., 2024a [63]	141–171 d	1–2 months		4–5 months		
Kirkpatrick et al., 2008 [33]	190 d	67 d in the early-vaccination group	167 d in the late-vaccination group		190 d, at weaning, in both groups	
Powell et al., 2012 [30]	Mean age = 209 ± 17 d	62 ± 17 d in the early-vaccination group	188 ± 17 d in the traditional-vaccination group		Mean 209 ± 17 d, at weaning, in both groups	

Note: Ages appearing in separate priming columns can represent alternative study groups rather than sequential doses administered to the same calves. For Erickson et al. (2021), calves were primed at either 24 h or 48 d, depending on treatment group; only groups primed at 24 h received a booster at 48 d. A blank cell indicates that no dose was administered at that stage. Some studies appear in both Supplementary Materials 3a and 3b because they reported clinical outcomes during both the pre-weaning and post-weaning periods.

References

34. Erickson, N.; Ellis, J.; Waldner, C.; Lardner, H.; Gow, S.; Campbell, J.; Berenik, A. A field comparison of heterologous and homologous routes of administration of modified live vaccines for the control of bovine respiratory disease in recently weaned beef calves. Can. Vet. J. 2020a, 61(5), 530–533. PMID: 32355354; PMCID: PMC7155885.

28. Erickson, N.; Ellis, J.; Waldner, C.; Lardner, H.; Gow, S.; Campbell, J.; Berenik, A. Comparison of bovine viral diarrhea virus-specific antibody responses of young beef calves vaccinated with either modified live virus or inactivated virus regimens. Can. Vet. J. 2020b, 61, 871–874. PMID: 32741994; PMCID: PMC7350141.

29. Erickson, N.E.; Berenik, A.; Lardner, H.; Lacoste, S.; Campbell, J.; Gow, S.; Waldner, C.; Ellis, J. Evaluation of bovine respiratory syncytial virus (BRSV) and bovine herpesvirus (BHV) specific antibody responses between heterologous and homologous prime-boost vaccinated western Canadian beef calves. Can. Vet. J. 2021, 62(1), 37–44. PMID: 33390597; PMCID: PMC7738673.

63. Erickson, N.E.N.; April, S.; Campbell, J.R.; Homerosky, E.; Ware, T.; Dorin, C.; Waldner, C.L.; Ellis, J.A. Comparison of postweaning bovine respiratory disease treatment rates between non-vaccinated control beef calves and calves variably primed and boosted using commercially available bovine coronavirus vaccines. Can. Vet. J. 2024a, 65(6), 581–586. PMID: 38827595.

50. Erickson, N.E.; Ware, T.; Campbell, J.; Larson, K.; Ellis, J.A.; Waldner, C.L. Comparison of pre-weaning bovine respiratory disease treatment rates between non-vaccinated control and variably primed and boosted beef calves receiving commercially available bovine coronavirus vaccines. Can. Vet. J. 2026, 67(2), 188–197.

33. Kirkpatrick, J.G.; Step, D.L.; Payton, M.E.; Richards, J.B.; McTague, L.F.; Saliki, J.T.; Confer, A.W.; Cook, B.J.; Ingram, S.H.; Wright, J.C. Effect of age at the time of vaccination on antibody titers and feedlot performance in beef calves. J. Am. Vet. Med. Assoc. 2008, 233(1), 136–142. doi:10.2460/javma.233.1.136; PMID: 18593324.

30. Powell, J.G.; Richeson, J.T.; Kegley, E.B.; Coffey, K.P.; Erf, G.F.; Brown, A.H.; Downum, W.; Ensley, D.T. Immunologic, health, and growth responses of beef calves administered pentavalent modified-live virus respiratory vaccine in the presence of maternal antibody versus a traditional vaccination regimen. Bovine Pract. 2012, 46(2), 122–30. doi:10.21423/bovine-vol46no2p122-130.
