# Supplementary material for: The Impact of Vaccination Practices in Cow–Calf Operations on Bovine Respiratory Disease Morbidity and Mortality in Beef Calves: A Rapid Scoping Review
Source: Animals (Basel). 2026 Nov 1;16(21):3454. doi: 10.3390/ani16213454 (PMC13761806; doi:10.3390/ani16213454)
Supplement: Supplementary file 1 [file animals-16-03454-s001.zip › SupplementaryMaterialS4.pdf]

Impact of vaccination practices in cow-calf operations on bovine respiratory disease morbidity and mortality in beef calves: a rapid scoping review

V. Margarita Sanguinetti, Nathan E.N. Erickson, Barbara Wilhelm, Colleen Pollock, Su Hyun Lim, Lianne McLeod, Cheryl L. Waldner

Supplementary Material S4. Descriptive summary of findings of 10 captured experimental challenge trials reporting viral detection or shedding (complete table). A condensed summary of these findings is presented as Table 3 in the main text.

Citation	Age at vaccination (age at challenge)	Study arms (calves per arm)	Sampling time (sample type)	Estimate type (groups)	Calves positive, n/N or % (CI or P value)	Pathogen load (CI or P value)
**BVDV challenge studies**						
Chamorro et al., 2015 [70]	Median = 72 d (117 d)	C = Saline (n = 12 crossbred steers); T1 = BRD Shield (n = 12); T2 = Express 5 (n = 12); T3 = Bovi-Shield Gold 5 (n = 12)	Day 6 (WBC, Serum)	Comparative (C, T1, T2, T3)	Proportion positive: C = 58.3%, T1 = 8.3%, T2 = 8.3%, and T3 = 36.4%; T1 and T2 significantly < C (P < 0.05); T3 not significantly different from C	NA
			Day 8, 10, 14 (WBC and Serum)	Comparative (C, T1, T2, T3)	Proportion positive in T1, T2, T3 significantly < C (*P* < 0.05). Magnitude not reported	NA
			Entire study period = 28 d (WBC and Serum)	Comparative (C, T1, T2, T3)	Calves viremic at least once over Days 0–28: C = 11/12 (91.7%), T1 = 2/12 (16.7%), T2 = 3/12 (25.0%), and T3 = 6/11 (54.5%). C and T3 had higher proportions than T1 and T2 (*P* < 0.05).	NA
			Entire study period (Nasal Swab, viremic calves)	Comparative (C, T1, T2, T3)	Viremic calves that shed virus over Days 0–28: C = 8/11 (72.7%), T1 = 0/2 (0%), T2 = 0/3 (0%), and T3 = 2/6 (33.3%). C was significantly higher than T1, T2, and T3 (*P* < 0.05).	NA
Erickson et al., 2023 [71]	24 hr, mean = 54 d (SD = 3) (6 mo, after abrupt weaning)	T1 = Bovi-Shield Gold 5 IN at ~24 hr, Triangle 5 + One Shot at 54 d (n = 9); T2 = Bovi-Shield Gold 5 IN at ~24 hr, Inforce 3 + One Shot BVD at 54 d (n = 9)	Entire 14-day study period (Buffy coat)	Comparative (T1, T2)	NA	Viremia was detected in both treatment groups (T1, T2).
			Day 7, 10, 14 (Buffy coat)	Comparative (T1, T2)	NA	T1 had significantly (*P* < 0.01) greater mean BVDV

Citation	Age at vaccination (age at challenge)	Study arms (calves per arm)	Sampling time (sample type)	Estimate type (groups)	Calves positive, n/N or % (CI or P value)	Pathogen load (CI or P value)
						concentration Day 7, 10, and 14 relative to T2. Magnitude not reported
			Entire study period (Buffy coat)	Descriptive	NA	T1 magnitude of viremia increased between Days 0 and 3, and between Days 3 and 7 (*P* = 0.05 and *P* = 0.01, respectively); remained unchanged between Days 7 and Day 10 (*P* = 0.70); and decreased between Days 10 and 14 (*P* = 0.03). Magnitudes not reported.
			Entire study period (Buffy coat)	Descriptive	NA	T2 did not have an increase in viremia until Day 7 (*P* = 0.01), and had a decrease at the next measurement point, Day 10 (*P* = 0.01). Magnitude not reported.
**BRSV challenge studies**						
Ellis et al., 2018 [72]	24 hr, 2 mo (Weaned at 6-7 mo, challenged day of weaning)	C = no Inforce 3 at birth, within 24 hr of birth, SC Bovi-Shield Gold FP5 at 2 mo (n = 2 Angus cross heifer calves); T1 = Inforce 3 at birth, Bovi-Shield Gold FP5 injectable at 2 mo (n = 9); T2 = Inforce 3 at birth, Triangle 5 SC at 2 mo (n = 9)	Day 0 (Deep Nasal Swab)	Descriptive	Proportion positive: C = T1 = T2 = 0	None of the calves had detectable BRSV on nasal swabs on the day of weaning/challenge (< 10 PFU/mL).
			Day 5 (Deep Nasal Swab)	Comparative (T1, T2)	Proportion positive: T1 = 6/9, T2 = 5/9 ("similar")	T1 = 30, range 30-380 PFU/mL; T2 = < 10, range 10-250 PFU/mL
			Day 5 (Deep Nasal Swab)	Descriptive	Proportion positive: C = 2/2	C1 calf = 360 PFU/mL, C2 = 340 PFU/mL
			Day 7 (Deep Nasal Swab)	Comparative (T1, T2)	Proportion positive: T1 = T2 = 1/9	T1 calf = 30 PFU/mL, T2 = 20 PFU/mL
			Day 7 (Deep	Descriptive	Proportion positive: C = 2/2	C1 calf = 40 PFU/mL, C2 =

Citation	Age at vaccination (age at challenge)	Study arms (calves per arm)	Sampling time (sample type)	Estimate type (groups)	Calves positive, n/N or % (CI or P value)	Pathogen load (CI or P value)
			Nasal Swab)			30 PFU/mL
			Study period (Deep Nasal swab)	Comparative (T1, T2)	Proportion positive: nonsignificant difference between T1, T2	Non-significant difference between T1, T2
Martínez et al., 2023 [31]	Within 6 hr of birth (Abruptly weaned at approximately 3 mo of age, challenge 5 d later)	C = saline IN (n = 25 bull calves); T1 = Inforce 3 IN (n = 25 bull calves)	Days 4-10 period (Nasal Secretions)	Comparative (C, T1)	Proportion positive: C = T1 = 100%	NA
			Study period = 28 d (Nasal Secretions)	Comparative (C, T1)	NA	Median Cts: Non-significant difference C/T1. Interquartile range: Non-significant difference C/T1
			Day 0, 14, 21, 28 (Nasal Secretions)	Comparative (C, T1)	Proportion positive: C = T1 = 0	NA
			Study period = 28 d (Nasal Secretions)	Comparative (C, T1)	Risk of shedding: (*P* = 0.60) C vs T1. Magnitude not reported.	NA
			Study period = 28 d (Nasal Secretions)	Comparative (C, T1)	Median number of days calves positive: C = 2.17 d, T1 = 2.36 d (*P* = 0.84)	NA
Martínez et al., 2022 [32]	Cows 6.5-7.5 months pregnant (Calves abruptly weaned at ~3 mo of age, challenge 5 d later)	C = 2 doses saline injection 21 d apart at 6.5–7.5 months of gestation (n = 20 steer calves born to unvaccinated dams); T1 = 2 doses Triangle 10 21 d apart at 6.5–7.5 months of gestation (n = 20 steer calves born to vaccinated dams)	Study period = 28 d (Nasal Secretions)	Comparative (C, T1)	Proportion positive: C = 4/20 and T1 = 1/20. These counts are descriptive; the reported inferential comparison concerned the probability of shedding over time.	NA
			Study period = 28 d (Nasal Secretions)	Comparative (C, T1)	Risk of shedding: Risk was significantly (*P* < 0.01) lower T1 relative to C. Magnitude not reported.	NA
			Study period =	Comparative	Median number of days	NA

Citation	Age at vaccination (age at challenge)	Study arms (calves per arm)	Sampling time (sample type)	Estimate type (groups)	Calves positive, n/N or % (CI or P value)	Pathogen load (CI or P value)
			28 d (Nasal Secretions)	(C, T1)	calves positive: C = 2 d, T1 = 1 d	
**BoHV-1 challenge studies**						
Hill et al., 2019 [51]	3–6 weeks; 5–6 months (abruptly weaned and transported at 5–6 months; second vaccination administered 1 day after weaning; challenged 4 days after the second vaccination)	C = IN saline 3 weeks, IN saline weaning (n = 10 Angus cross calves); T1 = IN Vista 5 SQ 3 weeks, IN saline weaning (n = 10); T2 = IN Vista 5 SQ 3 weeks, IN Vista 5 SQ weaning (n = 10); T3 = IN saline 3 weeks, IN Vista 5 SQ at weaning (n = 10); T4 = IN Vista 5 SQ 3 weeks, SC Vista 5 SQ weaning (n = 10)	Day 3 (Nasal Swabs)	Comparative (C, T1, T2, T3, T4)	NA	C had significantly increased shedding Day 3 relative to T1-4 (*P* < 0.01). Magnitude not reported
			Study period = 12 d (Nasal Swabs)	Comparative (C, T1)	NA	T1 relative to C, nonsignificant decrease in shedding if single IN vaccination at 3 weeks. Magnitude not reported.
			Study period = 12 d (Nasal Swabs)	Comparative (C, T3)	NA	T3 relative to C, significant (*P* < 0.01) decrease in shedding if single IN vaccination at 6 mo. Magnitude not reported.
			Study period = 12 d (Nasal Swabs)	Comparative (C, T2)	NA	T2 relative to C, significant decrease in shedding if IN + IN booster. Magnitude not reported.
			Study period = 12 d (Nasal Swabs)	Comparative (C, T4)	NA	T4 relative to controls, nonsignificant decrease in shedding if IN priming with SC booster
			Study period = 12 d (Nasal Swabs)	Comparative (T2, T3)	NA	Significant reduction in shedding (*P* < 0.01) greater for calves boosted IN at 6 mo relative to primed IN at 6 mo. Magnitude not

Citation	Age at vaccination (age at challenge)	Study arms (calves per arm)	Sampling time (sample type)	Estimate type (groups)	Calves positive, n/N or % (CI or P value)	Pathogen load (CI or P value)
						reported.
**Combined viral challenge studies**						
Chamorro et al., 2016 [69] (Challenge targets: BVDV, BoHV-1)	Median 95 d, Range 60-128 d (Challenge initiated 45 d after vaccination; median age approximately 140 d)	C = Saline injection (n = 11); T1 = BRD Shield (n = 11); T2 = Express 5 (n = 10); T3 = Bovi-Shield Gold 5 (n = 11); T4 = Viralign 6 (n = 11)	Day 0 (All samples: Serum, WBCs, Deep Nasal Swabs)	Descriptive (C, T1-4)	BVDV: Number positive = 0, all groups	NA
			Day 3-11 (Serum or WBCs)	Descriptive (C)	BVDV: Proportion positive = 9/11	NA
			Day 6 (Serum or WBCs)	Descriptive (T1)	BVDV: Proportion positive = 1/11	NA
			Day 14 (Serum or WBCs)	Descriptive (T2)	BVDV: Proportion positive = 2/10	NA
			Day 6-10 (Serum or WBCs)	Descriptive (T3)	BVDV: Proportion positive = 4/11	NA
			Study period = 28 d (Serum or WBCs)	Descriptive (T4)	BVDV: Number positive = 0	NA
			Day 6, 7, 8, 9, 10, 14 (Serum or WBCs)	Comparative (C, T1, T2, T4)	BVDV: Proportion positive. The proportion positive was significantly higher in C than in T2 and T4	NA
			Day 6, 7, 8, 9, 10, 14 (Serum or WBCs)	Comparative (C, T2)	BVDV: Proportion positive. The proportion positive was significantly higher in C than in T2	NA
			Day 10, 14 (Serum or WBCs)	Comparative (C, T4)	BVDV: Proportion positive. The proportion positive was significantly higher in C than in T4	NA
			Days 6-21 (Deep Nasal Swab)	Descriptive (C)	BVDV: Proportion positive = 5/11	NA
			Day 9, 28 (Deep	Descriptive	BVDV: Proportion positive	NA

Citation	Age at vaccination (age at challenge)	Study arms (calves per arm)	Sampling time (sample type)	Estimate type (groups)	Calves positive, n/N or % (CI or P value)	Pathogen load (CI or P value)
			Nasal Swab)	(T1)	= 2/11	
			Day 8 (Deep Nasal Swab)	Descriptive (T2)	BVDV: Proportion positive = 1/10	NA
			Day 8, 9, 21 (Deep Nasal Swab)	Descriptive (T3)	BVDV: Proportion positive = 3/11	NA
			Study period (Deep Nasal Swab)	Descriptive (T4)	BVDV: Number positive = 0	NA
			Days 6-21 (Deep Nasal swab)	Comparative (C, T1-3)	BVDV: Proportion positive nonsignificant difference	NA
			Day 9, 28 (Deep Nasal Swab)	Comparative (C, T4)	BVDV: Number of shedders C significantly > number of shedders T4 (*P* = 0.01). Magnitude not reported.	NA
			Day 0 (All samples: Serum, WBCs, Deep Nasal Swabs)	Descriptive (C, T1-4)	BoHV-1: Number positive = 0, all groups	NA
			Study period (Serum)	Descriptive (C, T1-4)	BoHV-1: Number positive = 0, all groups	NA
			Day 7, 9, 14 (Deep Nasal Swab)	Descriptive (C)	BoHV-1: Number positive = 1/11 each day	NA
			Day 6, 9 (Deep Nasal Swab)	Descriptive (T1)	BoHV-1: Number positive = 3/11	NA
			Day 9, 14 (Deep Nasal Swab)	Descriptive (T2)	BoHV-1: Number positive = 1/10	NA
			Day 3, 7 (Deep Nasal Swab)	Descriptive (T3)	BoHV-1: Number positive = 2/11	NA
			Day 7 (Deep Nasal Swab)	Descriptive (T4)	BoHV-1: Number positive = 1/11	NA
			Study period (Deep Nasal Swab)	Comparative (C, T1-4)	Proportion positive nonsignificantly different across all groups.	NA
Erickson et al., 2024b [67] (Challenge	Birth and 44 d (challenged approximately 1	T1 = Calf-Guard and Inforce 3 IN at birth, ScourGuard 4KC +	Day 0 (Nasal Swabs)	Descriptive (T1, T2)	BCoV: Number positive nonsignificant difference T1 vs T2	NA

Citation	Age at vaccination (age at challenge)	Study arms (calves per arm)	Sampling time (sample type)	Estimate type (groups)	Calves positive, n/N or % (CI or P value)	Pathogen load (CI or P value)
targets: BCoV, BPIV-3, BRSV)	day after abrupt weaning at 169 d [SD = 5 d])	Triangle 5 (i.e., KV group) at 44 d (n = 9); T2 = Calf-Guard and Inforce 3 IN at birth, Calf-Guard and Bovi-Shield Gold FP5 (MLV) at 44 d (n = 11)				
			Days 5-6 (Nasal Swabs)	Comparative (T1, T2)	NA	BCoV: T1 (0.3 times, $P = 0.02$) or T2 (0.2 times, $P < 0.01$) groups lower Day 6 vs. Day 5
			Day 10 (Nasal Swabs)	Comparative (T1, T2)	NA	BCoV: count was 0.2 times the count ($P = 0.05$) for IN-MLV than for IN-KV
			Day 0 (Nasal Swabs)	Descriptive (T1, T2)	NA	BPIV-3: T1 = T2 = 0
			Day 6, 10 (Nasal Swabs)	Descriptive (T1)	NA	BPIV-3: T1 BPIV-3 0.2 times on Day 10 versus Day 6 ($P = 0.01$)
			Days 5-6, Days 6-10 (Nasal Swabs)	Descriptive (T2)	NA	BPIV-3: T2 had higher counts of BPIV-3 than the IN-KV group on Day 6 (9.6 times, $P = 0.02$) and Day 10 (32.8 times, $P = 0.04$)
			Day 0 (Nasal Swabs)	Descriptive (T1, T2)	NA	BRSV: T1 = T2 = 0
			Days 5-6 (Nasal Swabs)	Descriptive (T1)	NA	BRSV: T1 count was lower ($P = 0.01$) on Day 6 relative to Day 5
			Days 5-6 (Nasal Swabs)	Descriptive (T2)	NA	BRSV: T2 count was higher ($P = 0.01$) on Day 6 than Day 5
			Days 6-10 (Nasal Swabs)	Descriptive (T1)	NA	BRSV: Day 10 BRSV counts were lower than on Day 6 for both IN-KV ($P = 0.002$) group
			Days 6-10 (Nasal Swabs)	Descriptive (T2)	NA	BRSV: Day 10 BRSV counts were lower than on Day 6 for both IN-MLV ($P = 0.01$)

Citation	Age at vaccination (age at challenge)	Study arms (calves per arm)	Sampling time (sample type)	Estimate type (groups)	Calves positive, n/N or % (CI or P value)	Pathogen load (CI or P value)
			Day 6, the day of maximal shedding (Nasal Swabs)	Comparative (T1, T2)	NA	On Day 6, the BRSV count in the IN-MLV group was 79.0 times that in the IN-KV group (*P* < 0.01)
Erickson et al., 2025 [49] (Challenge targets: BRSV, BPIV-3)	Birth, 39 ± 2 d (160 d, day after abrupt weaning at 159 ± 2 d)	T1 = (prime-boost = PRB) Inforce 3 at birth, 39 ± 2 d, Bovi-Shield Gold One Shot SC (n = 9); T2 = (INJ) No vac at birth, SC (INJ) 39 ± 2 d, Bovi-Shield Gold One Shot (n = 9)	Day 5 (Nasal Swabs)	Comparative (T1, T2)	NA	BRSV: T2 had counts 2 times greater than T1 (*P* < 0.01)
			Day 7 (Nasal Swabs)	Comparative (T1, T2)	NA	BRSV: T2 had counts 3.4 times greater than T1 (*P* < 0.01)
			Day 5 (Nasal Swabs)	Comparative (T1, T2)	NA	BPIV-3: T2 had counts 2.3 times greater than T1 (*P* < 0.01)
			Day 7 (Nasal Swabs)	Comparative (T1, T2)	NA	BPIV-3: T2 had counts 17.1 times greater than T1 (*P* < 0.01)
Martínez et al., 2024 [48] (Challenge targets: BRSV, BoHV-1)	2 mo, 6 mo (3 d post-weaning at ~6 mo of age)	C = saline at 2 mo, saline at 6 mo; T1 = Bovilis Vista 5 SQ at 2 mo, IN Nasalgen 3 at 6 mo; T2 = saline at 2 mo, IN Nasalgen 3 at 6 mo	Study period = 28 d (Nasal Swabs)	Comparative (C, T1, T2)	Proportion positive: At every sampling day, a lower proportion of calves were positive to BRSV relative to BoHV-1, across all groups	NA
			Day -2 (Nasal Swabs)	Comparative (C, T1, T2)	Proportion positive BRSV: C = T1 = T2 = 2/10	NA
			Study period = 30 d, i.e., Day -2 to Day 28 (Nasal Swabs)	Comparative (C, T1, T2)	Proportion positive BRSV: Non-significant difference across sampling days.	NA
			Day -2 (Nasal Swabs)	Descriptive (C)	Proportion positive BoHV-1 = 7/10	NA
			Day -2 (Nasal Swabs)	Descriptive (T1)	Proportion positive BoHV-1 = 8/10	NA
			Day -2 (Nasal Swabs)	Descriptive (T2)	Proportion positive BoHV-1 = 7/10	NA
			Day -2, 5, 7, 10, 21, 28 (Nasal	Comparative (C, T1, T2)	Proportion positive BoHV-1 was significantly lower Day	NA

Citation	Age at vaccination (age at challenge)	Study arms (calves per arm)	Sampling time (sample type)	Estimate type (groups)	Calves positive, n/N or % (CI or P value)	Pathogen load (CI or P value)
			Swabs)		21, 28 relative to Day -2, 5, 7, 10	
			Study period = 30 d, i.e., Day -2 to Day 28 (Nasal Swabs)	Comparative (C, T1, T2)	Day -2 to 28, the risk of shedding BRSV and BoHV-1 was not significantly different across time.	NA

Where original publications reported significance testing but did not provide effect-size estimates, magnitude was recorded as not reported.

Note: For Erickson et al., 2025 [49], 22 calves were initially recruited, but 4 were excluded/lost to follow-u*p* prior to the viral challenge phase, resulting in 18 calves (9 per arm) analyzed for secondary outcomes. For Chamorro et al., 2015 [70], percentages were recalculated from the reported counts, and T3 included 11 calves at challenge because one calf was euthanized before challenge.

Commercial vaccines named in the table (antigens; vaccine type): BRD Shield, Express 5, Bovi-Shield Gold 5, and Bovi-Shield Gold FP5 (BoHV-1, BVDV-1, BVDV-2, BPIV-3, and BRSV; MLV); Bovi-Shield Gold One Shot (the same five viruses, MLV, plus *M. haemolytica* toxoid); Bovilis Vista 5 SQ (BoHV-1, BVDV-1, BVDV-2, BPIV-3, and BRSV; MLV; given IN and SC by Hill et al., 2019 [51]); Viralign 6 (BoHV-1, BVDV-1a, BVDV-1b, BVDV-2, BPIV-3, and BRSV; MLV); Inforce 3 and Nasalgen 3 (BoHV-1, BPIV-3, and BRSV; MLV for intranasal use); Triangle 5 (BoHV-1, BVDV-1, BVDV-2, BPIV-3, and BRSV; KV); Triangle 10 (the same five viruses, KV, plus five *Leptospira* serovars); One Shot (*M. haemolytica* toxoid); One Shot BVD (BVDV-1 and BVDV-2, MLV, plus *M. haemolytica* toxoid); Calf-Guard (bovine rotavirus and BCoV; MLV); and ScourGuard 4KC (bovine rotavirus and BCoV, KV, plus *Escherichia coli* K99 bacterin and *Clostridium perfringens* type C toxoid).

Abbreviations

BCoV = Bovine coronavirus; BoHV-1 = Bovine herpesvirus-1; BPIV-3 = Bovine parainfluenzavirus-3; BRSV = Bovine Respiratory Syncytial Virus; BVDV = Bovine viral diarrhea virus; SD = Standard deviation; d = Calendar day; hr = hour; mo = month; C = Control group; T1, T2, ... = Treatment groups; CI = confidence interval; Ct = cycle threshold; IN = intranasal; SC = subcutaneous; MLV = modified-live virus; KV = killed (inactivated) virus; WBC = White Blood Cells; NA = Not applicable.

References

31. Martínez, D.A.; Chamorro, M.F.; Passler, T.; Huber, L.; Walz, P.H.; Thoresen, M.; Raithel, G.; Silvis, S.; Stockler, R.; Woolums, A.R. Local and systemic antibody responses in beef calves vaccinated with a modified-live virus bovine respiratory syncytial virus vaccine at birth following BRSV infection. Vet. Sci. 2023, 10(1), 20. doi:10.3390/vetsci10010020. PMID: 36669022; PMCID: PMC9863489.

32. Martínez, D.A.; Chamorro, M.F.; Passler, T.; Huber, L.; Walz, P.H.; Thoresen, M.; Raithel, G.; Silvis, S.; Stockler, R.; Woolums, A.R. The titers, duration, and residual clinical protection of passively transferred nasal and serum antibodies are similar among beef calves that nursed colostrum from vaccinated or unvaccinated dams and were challenged experimentally with bovine respiratory syncytial virus at three months of age. Am. J. Vet. Res. 2022, 83(11), 1–9. doi:10.2460/ajvr.22.07.0118. PMID: 36173761.

48. Martínez, D.A.; Chamorro, M.F.; Passler, T.; Huber, L.; Falkenberg, S.; Walz, P.H.; Thoresen, M.; Raithel, G.; Silvis, S.; Dimitrov, K.M.; Stockler, R.; Woolums, A.R. Intranasal booster vaccination of beef steers reduces clinical signs following experimental coinfection with BRSV and BHV-1 without reducing shedding of BRD-associated bacteria. Am. J. Vet. Res. 2024, 85(5), ajvr.23.11.0266. doi:10.2460/ajvr.23.11.0266.

49. Erickson, N.E.; Waldner, C.L.; Sniatynski, M.; Lacoste, S.; Moya, D.; Ellis, J.A. Comparing clinical and immunologic response efficacy of systemic prime versus mucosal prime and systemic boost vaccination of beef calves after coinfection with bovine respiratory syncytial virus and bovine parainfluenza virus type 3 at weaning. Can. Vet. J. 2025, 66(12), 1315–1325.

51. Hill, K.; Arsic, N.; Nordstrom, S.; Griebel, P.J. Immune memory induced by intranasal vaccination with a modified-live viral vaccine delivered to colostrum fed neonatal calves. Vaccine 2019, 37(51), 7455–7462. doi:10.1016/j.vaccine.2019.09.080. PMID: 31590936.

67. Erickson, N.; Waldner, C.; Snyder, E.; Araya, M.B.; Sniatynski, M.; Ellis, J. Comparative efficacy of modified-live and inactivated vaccines in boosting responses to bovine respiratory syncytial virus, bovine parainfluenza virus Type 3, and bovine coronavirus following neonatal mucosal priming of beef calves. Can. Vet. J. 2024b, 65, 791–801.

69. Chamorro, M.F.; Walz, P.H.; Passler, T.; Palomares, R.A.; Newcomer, B.W.; Riddell, K.P.; Gard, J.; Zhang, Y.; Galik, P. Efficacy of four commercially available multivalent modified-live virus vaccines against clinical disease, viremia, and viral shedding in early-weaned beef calves exposed simultaneously to cattle persistently infected with bovine viral diarrhea virus and cattle acutely infected with bovine herpesvirus 1. Am. J. Vet. Res. 2016, 77(1), 88–97. doi:10.2460/ajvr.77.1.88. PMID: 26709942.

70. Chamorro, M.F.; Walz, P.H.; Passler, T.; van Santen, E.; Gard, J.; Rodning, S.P.; Riddell, K.P.; Galik, P.K.; Zhang, Y. Efficacy of multivalent modified-live virus vaccines administered to early-weaned beef calves subsequently challenged with virulent bovine viral diarrhea virus type 2. BMC Vet. Res. 2015, 11, 29. doi:10.1186/s12917-015-0342-8. PMID: 25880998; PMCID: PMC4334402.

71. Erickson, N.; Godson, D.; Gow, S.; Lacoste, S.; Waldner, C.; Ellis, J. Comparative efficacy of modified-live and inactivated vaccines in boosting disease-sparing responses to bovine viral diarrhea virus challenge in neonatally mucosally primed weanling beef calves. Can. Vet. J. 2023, 64, 321–328. PMID: 37008637; PMCID: PMC10031784.

72. Ellis, J.; Gow, S.; Berenik, A.; Lacoste, S.; Erickson, N. Comparative efficacy of modified-live and inactivated vaccines in boosting responses to bovine respiratory syncytial virus following neonatal mucosal priming of beef calves. Can. Vet. J. 2018, 59, 1311–1319.
