# Supplementary material for: The Impact of Vaccination Practices in Cow–Calf Operations on Bovine Respiratory Disease Morbidity and Mortality in Beef Calves: A Rapid Scoping Review
Source: Animals (Basel). 2026 Nov 1;16(21):3454. doi: 10.3390/ani16213454 (PMC13761806; doi:10.3390/ani16213454)
Supplement: Supplementary file 1 [file animals-16-03454-s001.zip › SupplementaryMaterialS5-PRISMA-ScRChecklist.pdf]

PRISMA-ScR Checklist

Preferred Reporting Items for Systematic Reviews and Meta-Analyses extension for Scoping Reviews

Completed for: Impact of vaccination practices in cow-calf operations on bovine respiratory disease morbidity and mortality in beef calves: a rapid scoping review

Section	Item	PRISMA-ScR checklist item	Reported location and assessment rationale	Assessment
**TITLE**	**1. Title**	Identify the report as a scoping review.	Title page: title identifies the report as a rapid scoping review.	Fully complete
**ABSTRACT**	**2. Structured summary**	Provide a structured summary that includes, as applicable, background, objectives, eligibility criteria, sources of evidence, charting methods, results, and conclusions related to the review questions and objectives.	Abstract: structured Background, Methods, Results, and Conclusions; the Methods state the information sources, core eligibility criteria, and that data were charted in Excel and independently reviewed.	Fully complete
**INTRODUCTION**	**3. Rationale**	Describe the rationale for the review in the context of what is already known and explain why the questions or objectives are suited to a scoping review approach.	Introduction: prior reviews, intervention complexity, knowledge gaps, and rationale for mapping heterogeneous evidence.	Fully complete
**INTRODUCTION**	**4. Objectives**	State the review questions and objectives explicitly, with reference to their key elements, such as population or participants, concepts, and context.	Introduction: explicit research questions and primary and secondary objectives; Figure 1.	Fully complete
**METHODS**	**5. Protocol and registration**	State whether a protocol exists, where it can be accessed, and any registration information, if available.	Methods, "Protocol and registration": protocol location in the University of Saskatchewan Harvest repository and departures from the protocol.	Fully complete
**METHODS**	**6. Eligibility criteria**	Specify the characteristics of eligible sources of evidence, including years, language, and publication status, and provide a rationale.	Methods, "Eligibility criteria": population, interventions and comparators, outcomes, study designs, publication years, language, geography, and report characteristics.	Fully complete
**METHODS**	**7. Information sources**	Describe all information sources and the date the most recent search was executed.	Methods, "Information Sources, Search Strategy, and Data Management," and Supplementary Material 1: all databases, platforms, manual sources, search dates, and the most recent rerun are identified consistently.	Fully complete
**METHODS**	**8. Search**	Present the full electronic search strategy for at least one database, including limits, so it can be repeated.	Supplementary Material 1: complete electronic search strategies, limits, dates, platforms, and retrieval counts, including the Mycoplasma bovis search update.	Fully complete
**METHODS**	**9. Selection of sources of evidence**	State the process for selecting sources of evidence included in the scoping review.	Methods, "Screening and Selection Process": pilot testing, blinded independent screening of a random 20% title-and-abstract sample, independent dual assessment of a 50-report full-text pilot, subsequent single-reviewer screening of the remaining full-text reports, and conflict resolution.	Fully complete
**METHODS**	**10. Data charting process**	Describe how data were charted, whether forms were calibrated or tested, whether charting was independent or duplicated, and any process for obtaining or confirming data.	Methods, "Data Collection Process": the data-extraction form was reviewed during a 50-record pilot exercise; extraction and independent review procedures, conflict resolution, and the absence of investigator contact are reported.	Fully complete
**METHODS**	**11. Data items**	List and define the variables sought and any assumptions or simplifications.	Methods, "Outcomes" and "Data Collection Process"; Tables 1–4; Supplementary Materials 2, 3a, 3b, and 4. Outcome-classification assumptions and the post hoc calculation rule are stated.	Fully complete
**METHODS**	**12. Critical appraisal of individual sources of evidence (optional)**	If critical appraisal was performed, explain why, describe the methods, and state how the information was used in synthesis.	Not performed. The absence of formal critical appraisal is reported in the Discussion limitations.	Not required
**METHODS**	**13. Synthesis of results**	Describe the methods used to handle and summarize charted data.	Methods, "Data Synthesis": narrative synthesis and descriptive tables, grouped by intervention population, outcome period, study design, and disease conditions.	Fully complete
**RESULTS**	**14. Selection of sources of evidence**	Report the numbers screened, assessed for eligibility, and included, with reasons for exclusions at each stage, ideally using a flow diagram.	Results and Figure 3: 3,469 records identified, comprising 3,461 records retrieved through database searches and 8 additional records identified manually; 1,654 duplicate records removed; 1,815 records screened; 197 reports assessed at full text; data extraction attempted for 54 reports; 28 studies reporting primary clinical outcomes summarized; and 18 additional studies reporting secondary outcomes only mapped. Reasons for exclusion are reported.	Fully complete
**RESULTS**	**15. Characteristics of sources of evidence**	For each source, present the characteristics for which data were charted and provide citations.	Results, Tables 1–4; Supplementary Materials 2, 3a, 3b, and 4.	Fully complete
**RESULTS**	**16. Critical appraisal**	If critical appraisal was performed, present the results.	Not applicable. Critical appraisal was not performed.	Not required

	**within sources of evidence (optional)**			
**RESULTS**	**17. Results of individual sources of evidence**	For each included source, present the relevant charted data related to the review questions and objectives.	Results; Tables 1–4; Supplementary Materials 2, 3a, 3b, and 4.	Fully complete
**RESULTS**	**18. Synthesis of results**	Summarize or present the charting results as they relate to the review questions and objectives.	Results, organized by the four intervention-outcome questions and study design; Table 2 summarizes the clinical findings of the natural-disease randomized controlled trials, and Table 4 provides the integrated evidence summary and knowledge gaps.	Fully complete
**DISCUSSION**	**19. Summary of evidence**	Summarize the main results, including an overview of concepts, themes, and types of evidence, relate findings to the questions and objectives, and consider relevance to key groups.	Discussion (including a summary of evidence gaps in relation to the secondary objective) and Conclusions.	Fully complete
**DISCUSSION**	**20. Limitations**	Discuss limitations of the scoping-review process.	Discussion limitations: rapid-review methods, no formal appraisal, language and date restrictions, heterogeneity, observational confounding, multicomponent programs, low event counts, and limitations of post hoc aggregate-data comparisons.	Fully complete
**DISCUSSION**	**21. Conclusions**	Provide a general interpretation of the results with respect to the questions and objectives and discuss implications or next steps.	Conclusions: interpretation, reporting recommendations, study-design considerations, and research priorities.	Fully complete
**FUNDING**	**22. Funding**	Describe funding sources for the included sources of evidence and for the scoping review, and describe the funders' role.	Back Matter, "Funding," and Methods, "Data Collection Process": review funding, grant number, and the funder's role are reported. Funding sources for included studies were not charted because this information was considered outside the scope of the present rapid review.	Fully complete

Assessment categories: Fully complete = all required reporting elements were identified in the attached manuscript or supplementary materials; Partially complete = one or more requested elements were absent, unclear, or inconsistent; Not required = optional PRISMA-ScR item not undertaken.

Assessment summary: 20 items fully complete; 2 optional items not required (Items 12 and 16).

Notes: Items 12 and 16 are optional under PRISMA-ScR.

Source: Tricco AC, Lillie E, Zarin W, et al. PRISMA extension for scoping reviews (PRISMA-ScR): checklist and explanation. Ann Intern Med. 2018;169:467–473. doi:10.7326/M18-0850.
